# Soda-baited RNAi yeast insecticides as effective Attractive Targeted Sugar Baits (ATSBs) for mosquito control

**DOI:** 10.1371/journal.pntd.0014319

**Published:** 2026-09-21

**Authors:** Akilah T. M. Stewart, Majidah Hamid-Adiamoh, Teresia M. Njoroge, Longhua Sun, Nikhella Winter-Reece, Rachel Shui Feng, Satish Singh, Lester D. James, Britton Sofhauser, Carolina Dille, Azad Mohammed, David W. Severson, Molly Duman-Scheel

**Affiliations:** 1 Department of Medical and Molecular Genetics, Indiana University School of Medicine, South Bend, Indiana, United States of America; 2 Eck Institute for Global Health, University of Notre Dame, Notre Dame, Indiana, United States of America; 3 Department of Life Sciences, Faculty of Science and Technology, The University of the West Indies, St. Augustine Campus, St. Augustine, Trinidad and Tobago; 4 College of Arts and Letters, The University of Notre Dame, Notre Dame, Indiana, United States of America; 5 Department of Biological Sciences, The University of Notre Dame, Notre Dame, Indiana, United States of America; University of California Davis School of Veterinary Medicine, UNITED STATES OF AMERICA

## Abstract

**Background:**

Attractive Targeted Sugar Baits (ATSBs) are a promising, environmentally friendly approach for mosquito control, but the direct field application, scalability and long-term effectiveness of ATSBs across diverse mosquito species remain significant challenges.

**Methodology/Principal findings:**

We assessed the efficacy of a genetically engineered RNA interference (RNAi) yeast strain (Sh.463) formulated in three sugar baits: soda (Coca-Cola), 10% sucrose, and a commercial mosquito bait (BaitStab), on *Aedes*, *Anopheles* and *Culex* mosquitoes. All RNAi yeast bait formulations induced significantly higher mean mortality (87–100%) compared to the control groups (0–9%), but RNAi yeast-soda baits resulted in significantly higher mortality rates of 94–100% recorded across all mosquito species. Additionally, to assess the competitiveness of the RNAi yeast-soda bait to other tropical sugar sources, semi-field choice assays were conducted in Trinidad, West Indies using competing flowering plants and fruits typically found in residential environments. The RNAi yeast-soda ATSB continued to perform well in the presence of many competing floral and fruit sugar sources during both *Aedes albopictus* and *Culex quinquefasciatus* trials, but the presence of several fruits and flowers did significantly reduce Sh.463-induced mosquito mortality with the degree of competitive deterrence being heavily modulated by mosquito species, sex, and floral resource, suggesting that further field testing will be necessary. The residual activity of the Sh.463 + soda formulation was retained for at least 14 months, with sustained 100% mortality in *C. quinquefasciatus* and 93–100% mortality in *Aedes aegypti*, *Anopheles gambiae* and *Anopheles stephensi*. The RNAi yeast-soda ATSB also performed well in semi-field studies performed with a prototype soda bottle feeder.

**Conclusions/Significance:**

This study demonstrates the potential of soda-baited RNAi yeast as a potent, long-lasting, and scalable platform for ATSB-based mosquito control as a component of integrated vector management programs.

## Author summary

Mosquito-borne diseases continue to affect millions of people worldwide, and current mosquito control methods face challenges such as low public uptake, insecticide resistance and environmental concerns. Here we evaluated a new and environmentally friendly approach to mosquito control using ATSBs. We tested genetically engineered species-specific yeast producing RNAi molecules capable of killing mosquitoes that feed on it. We mixed the yeast with three different sugar baits, including soda, 10% sucrose, and the commercial mosquito bait BaitStab formulation, and evaluated how well they worked against different mosquitoes. The results showed that the RNAi yeast mixed with soda was the most effective, killing up to 100% of mosquitoes in laboratory and outdoor tests. The bait remained effective in the presence of many competing natural tropical fruit and floral sugar sources. Remarkably, the bait, which can be delivered in a soda bottle feeder, stayed active for at least 14 months under simulated field conditions. These findings suggest that soda-based RNAi yeast baits could provide a practical, long-lasting and scalable tool for mosquito control and may help strengthen future strategies to reduce mosquito-borne diseases.

## Introduction

Mosquito-borne diseases pose significant threats to global health, accounting for nearly one million deaths annually worldwide [1]. The primary strategy for preventing these diseases is mosquito control using synthetic insecticides. However, the long-term effectiveness of this approach is challenged by low adherence to control measures, increasing instances of insecticide resistance, and growing concerns about the harmful effects of insecticides on non-target organisms [2,3]. Addressing these challenges is crucial for ensuring the effectiveness and sustainability of the current strategies. The success of the World Health Organization (WHO) global plan for insecticide resistance management [4] relies on the effective implementation of new insecticides [3]. Hence, extensive research efforts are underway to develop novel active ingredients with alternative modes of action, as well as innovative approaches such as biological control, genetic modification, and behaviorally based interventions to reduce reliance on chemical insecticides and enhance the sustainability of vector control programs [5].

RNA interference (RNAi) is an emerging and promising approach with significant potential for mosquito control. Our recent studies [6–11] have demonstrated that RNAi pesticides, which can be inexpensively produced in yeast, can silence genes required for mosquito survival. These novel pesticides are designed specifically to target conserved sequences in mosquitoes that are not found in non-target organisms. The pesticides are delivered as oral insecticides using heat-inactivated lyophilized yeast as a carrier, and the lethal yeast is capable of effectively killing mosquito vectors, including *Aedes*, *Culex* and *Anopheles spp.,* without any impact on non-target organisms [6–11].

ATSBs, a new paradigm for vector control, capitalize on the natural sugar feeding behavior of female and male mosquitoes that are drawn to feed on a sugar bait mixed with an insecticide. ATSB-mediated delivery of several non-specific insecticides has been successfully applied for direct control of *A. aegypti*, *A. albopictus* and other *Aedes* species [12–19] both in laboratory and field conditions. Similarly, *Culex pipiens, C. quinquefasciatus, C. tarsalis* and a variety of other *Culex* species, including nuisance biters, have been successfully targeted with ATSBs [13,20–23]. In addition, ATSBs are also showing great promise for targeting *Anopheles* species [24–26]. In field trials, ATSBs reduced the density of *Anopheles* mosquitoes [24–26]. However, the evolution of resistance mechanisms remains a significant challenge for the use of ATSBs, underscoring the need to identify second-generation ATSB insecticides. Moreover, despite the addition of protective barriers to bait stations and efforts to limit ATSB applications to non-flowering vegetation, it is difficult to completely eliminate risks to pollinators and other non-target organisms [27].

Given that the success of ATSB deployment relies on effectively diverting mosquitoes from natural sugar sources, it is critical that baits which are highly attractive to mosquitoes are used. We have developed a novel mosquito-specific yeast insecticide, Sh.463 [8], that expresses interfering RNA which targets a conserved site in the *Shaker (Sh)* gene of multiple species of mosquitoes, but not humans or other non-target organisms, including honeybees. This eco-friendly universal mosquito insecticide has performed well in lab and semi-field trials [6,8,28], promoting increased sugar feeding rates and killing up to 100% of mosquitoes when deployed using the commercial BaitStab matrix. However, when ATSBs prepared with BaitStab and the broad-based chemical insecticide dinotefuran were evaluated in recent Phase III clinical trials [29], despite the initially promising entomological impacts of these ATSB baits [26], significant epidemiological impacts were ultimately not observed [29]. It is also presently unclear if the bait deployed in the trial, which assessed malaria transmission, will be the most effective lure for *Aedes* and *Culex* arboviral vector mosquitoes in the field.

Building on our initial data from the Sh.463 [8] RNAi yeast strain, which targets the mosquito *Sh* gene, the addition of which may increase the attractiveness of BaitStab [26] in both laboratory [6] and simulated semi-field conditions [28], this study aimed to further enhance the effectiveness of the yeast by identifying a more attractive sugar bait (ASB) formulation for use in Sh.463-based ATSB stations targeting multiple mosquito species. We hypothesized that incorporating soda-based sugar formulations with the Sh.463 RNAi yeast insecticide will significantly increase mosquito mortality across multiple mosquito species. This study assessed the efficacy of Sh.463 RNAi yeast with three sugar baits in *Aedes, Anopheles* and *Culex* mosquitoes. We further assessed the ATSB efficacy in semi-field conditions and against natural plant sugar sources available to mosquitoes in tropical environments.

## Methods

### Ethics statement

This study was approved by the Institutional Review Board of the University of the West Indies (UWI; Study CEC403/12/17), the Southwest Regional Health Authority, which is a division of the Trinidad and Tobago Ministry of Health, and by the head of the UWI St. Augustine Department of Life Sciences.

### Preparation of RNAi yeast

A previously developed broad-based, high-expression Sh.463 RNAi yeast strain [6] that expresses short hairpin RNA (shRNA) targeting conserved sequences in mosquito *Sh* genes across multiple mosquito species [8] was used in this investigation. The genotype of the Sh.463_56.10R yeast strain (referred to herein as Sh.463) is: *MATa*, *PiggyBac* (*leu2d/P*_*TDH3*_*-shRNA_463-T*_*CYC1*_, *P*_*TDH3*_*-shRNA_463-T*_*CYC1*_, *P*_*TDH3*_*-shRNA_463-T*_*CYC1*_), *CEN/ARS* (*URA3/SPBase-Sc-CO*) [6]. The control yeast strain (Control_347.1R, hereafter referred to as Control), which expresses a hairpin with no known target in any mosquito species, has the following genotype: *MATa, PiggyBac* (*LEU2/P*_*TDH3*_*-shRNA_Ctrl-T*_*CYC1*_), *2um* (*URA3/SPBase_Sc-CO*), *PiggyBac* (*HIS3/P*_*TDH3*_*-shRNA_Ctrl-T*_*CYC1*_), *CEN/ARS* (*URA3/SPBase_Sc-CO*) *PiggyBac* (*trp1d/P*_*TDH3*_*-shRNA_Ctrl-T*_*CYC1*_), *CEN/ARS* (*URA3/SPBase_Sc-CO*) [6]. The yeast was prepared, heat killed and lyophilized as described [30].

### Mosquito rearing for laboratory experiments

The mosquito strains used for all laboratory experiments included *A. albopictus* Gainesville (MRA-804, Biological and Emerging Infections (BEI) Research Resources Program, NIAID, NIH*), A. aegypti* Liverpool-IB12 (LVP-IB12), *A. gambiae* G3 (BEI Resources: MRA-112, contributed by Mark Q. Benedict), *A. stephensi* strain STE2 (MRA-128, BEI Resources), *C. quinquefasciatus* JHB (NR-43025, BEI Resources), and *C. pipiens* (developed from Niles, MI, USA ovitrap collection) [9]. Mosquitoes were reared in the insectary facility at the Indiana University School of Medicine, United States, under standard insectary conditions [31]: 27 ± 2 °C; 70 ± 10% relative humidity and a 12-h light/dark cycle.

### Preparation of sugar formulations

Three sugar delivery carriers with corresponding formulations were tested: 10% sucrose solution, BaitStab matrix without dinotefuran (Westham Co., Israel) and soda, each combined with either the Sh.463 or Control RNAi yeast. The 10% sucrose solution (ASB-Sugar) represents the standard sugar for maintaining mosquito colonies and was prepared by mixing 10 g of sucrose with 100 ml of distilled water. The BaitStab matrix [hereafter referred to as Westham bait (WH), acquired from Westham Co., Israel] is a proprietary sugar attractant [32] used in several multi-country ATSB field trials, with its effectiveness across these trials recently summarized [33]. In the current study, the bait was used without any dinotefuran. Aliquots of this bait were used directly without any modification to the original formulation. Coca-Cola (referred to herein as Soda), which contains approximately 11% sucrose, was selected as the soda-based attractive sugar bait (ASB) due to its high sugar content and proven efficacy in inducing significant mosquito feeding [34], including with *Aedes japonicus* [35]. Moreover, it is universally available and easily accessible as both an attractant and a sugar source for an ATSB. Apart from being degassed, the soda ASB was used in its original form without modification. Degassing was achieved by vigorously shaking the soda bottle through ten cycles, with the gas released by gently opening the lid at the end of each cycle. This degassing was critical for utility of the bait in the prototype feeder described (see below), as the feeder would leak when soda that had not been degassed was used.

### Mosquito yeast feeding experiments

Different experimental approaches were employed to assess mosquito feeding preferences using mortality rates and residual efficacy of the RNAi yeast in the three sugar baits. In all experiments except the fruit/flower competition assays discussed below, 25 non-blood-fed female mosquitoes (4–5 days old) were used. Prior to RNAi yeast feeding, mosquitoes were either sugar-starved for 24 hours for *A. gambiae* which does not tolerate longer starvation times due to their smaller body size and lower general energy reserves that render them less tolerant to prolonged nutritional starvation [36,37]. *A. albopictus, A. aegypti, C. quinquefasciatus* and *C. pipiens* were starved for 48 hours. Water was provided using cotton balls during starvation only for *A. gambiae* and *A. stephensi*. Mosquitoes were fed with the RNAi yeast mixture (Sh.463/Control + sugar bait) overnight, after which they were maintained on a 10% sucrose solution for six days when the trial was concluded. Mortality was monitored for six days following feeding. A total of nine biological replicates (three replicates per trial) were conducted for each sugar bait experiment.

All experiments performed with the lethal yeast Sh.463 strains were designated as ATSBs or Sh.463, whereas those using Control yeast were referred to as Control. In addition, experiments conducted with the individual sugar formulations alone, categorized as Attractive Sugar Baits or ASBs, were also performed. The combinations of RNAi yeast and sugar formulations used in all experiments are henceforth identified as follows:

i) Sh.463-Sugar: Sh.463 + 10% sugar
ii) Control-Sugar: Control + 10% sugar
iii) ASB-Sugar: 10% sugar alone
iv) Sh.463-WH: Sh.463 + WH
v) Control-WH: Control + WH
vi) ASB-WH: WH alone
vii) Sh.463-Soda: Sh.463 + soda
viii) Control-Soda: Control + soda
ix) ASB-Soda: soda alone

### Evaluation of mosquito mortality in the three RNAi yeast-sugar formulations

ATSB trials were conducted with individual sugar formulations inside circular (3.7L) mosquito cages mainly following the previously described protocol [7,8,10,11]. Briefly, a paste of sugar bait-yeast mixture containing 40 mg of lyophilized yeast and 120 μL of sugar bait (final concentration: 333.3 μg/μL), was prepared and fed to starved mosquitoes inside cages. Following initial optimization, the Sh.463-Sugar and ASB-Sugar experiments were presented to mosquitoes in petri dishes, whereas the Sh.463-WH and ASB-WH experiments were presented in a sealed miniature sachet feeder made with 5 and 25 micron food-grade nylon membranes (Amazon, Seattle Washington) and prepared as described [9]. The baits were administered using MUDUODUO automatic bird drinker cups (Amazon, Seattle Washington) custom-designed as ATSB feeders as described [38]. Briefly, a yeast paste containing 200 mg of either control or Sh.463 yeast in 300–360 µL of soda (556–667 µg/µL) was evenly spread inside a sachet made from the same food-grade nylon membrane used above. The sachet was then placed in the well of a MUDUODUO automatic bird feeder cup. A 12 oz Coca-Cola bottle was filled with 120 mL of degassed soda and inverted onto the feeder to provide a continuous supply of soda to the yeast inside the sachet. The membrane was secured in place using a wick made from a small piece of clean dehumidifier filter (Honeywell Home, Charlotte, NC). Mortality was recorded for six days post-yeast feeding.

### Dual-choice ATSB experiments

Dual-choice ATSB experiments were conducted to evaluate mosquito feeding preferences between different sugar bait formulations. The experiments were performed in 30 cm × 30 cm x 30 cm Bug Dorm cages (MegaView Science, Taiwan) using both Sh.463 ATSBs and the corresponding Control for each sugar formulation simultaneously. In each experimental cage, two baits were presented: one containing the Sh.463 and the other containing Control RNAi yeast in either of two sugar formulations. ASBs for each sugar type were also included in separate negative control cages.

Specifically, for the dual-choice experiments between soda and 10% sugar ASBs, Sh.463-Soda (Sh.463 + soda) and Control-Sugar (Control + 10% sugar) were placed in one cage, while Control-Soda (Control + soda) and Sh.463-Sugar (Sh.463 + 10% sugar) were placed in a separate cage. Similarly, in the soda versus WH comparison, the bait pairs included Sh.463-Soda and Control-WH in one cage, and Control-Soda and Sh.463-WH in another.

To assess sugar bait feeding preferences independent of mortality, dual-choice assays were conducted with 0.5% uranine and 0.5% rhodamine B dye-marked baits using the methodology described by Aviles et al. [39] and Sarkar et al. [40]. The study was performed with the Soda-Control yeast formulation vs. WH-Control yeast formulation. Eight replicate trials, each with 25 adult mosquitoes per cage (N = 200 total mosquitoes) were performed. Both the type of dye used as a tracer and the bait location were permutated in these assays, which were conducted on three-day-old adult female *C. pipiens* that were sugar-starved for 48 hours and subsequently allowed to feed on the ATSB yeast baits from petri dishes for 4 hours. Following feeding, mosquitoes were anaesthetized using ${\text{CO}}_{\text{2}}$ gas and examined using a transilluminator and fluorescence imaging to visually score ingestion and determine whether the dye-marked yeast had been successfully ingested.

For subsequent ATSB choice trials, all baits were positioned in opposite corners of the experimental cages to ensure equal exposure. To reduce positional access bias, the locations of the baits were again permutated in each replicate. The RNAi yeast-sugar mixtures were prepared at the same final concentration of 333.3 μg/μL as described above but at double the volume and applied according to the optimal delivery method for each formulation as described. Mosquitoes were fed overnight in the soda versus sugar trials. In the soda versus WH trials, the initial 12 hr feeding period was later reduced to four hours to prevent soda evaporation and to ensure that mortality could be induced by a single feeding. Each dual-choice pair was tested in three technical replicates, and mortality was also monitored for six days.

### Semi-field assessment of Sh.463-Soda in soda bottle feeders

To assess the most optimal bait from previous trials in ambient environmental conditions, experiments were also conducted in semi-field conditions following the Yeast-ATSB-Soda and ASB-Soda experiments described above and as previously reported [38]. The experiment was performed using *C. pipiens* females in September and October 2025 at the Indiana University School of Medicine. Three biological trials, each consisting of three technical replicates, were performed. The same feeders were used for all the trials over a four-week period. During the semi-field trial period (n = 27 days), the average daily temperature was 20.6 ± 5.2 °C and ranged from lows of 6.1 °C to highs of 41.1 °C. The average percentage relative humidity was 45.4 ± 15.2% and ranged from 25.5 to 93.8%. The average daily minimum percentage relative humidity was 45.7 ± 13.2%, and the average daily maximum percentage relative humidity was 79.5 ± 10.0%.

### Semi-field evaluation of mosquito attraction to Sh.463-Soda in the presence of competing natural plant sugar sources

Semi-field-based dual choice experiments were also conducted to determine whether the presence of local fruits and flowers impacted Sh.463-Soda efficacy. These experiments were carried out between May and August 2023 at the rooftop laboratory of the University of the West Indies, St. Augustine Campus in Trinidad, West Indies. The experimental flowers and fruits tested included *Allamanda cathartica* (yellow trumpet), *Catharanthus roseus* (Madagascar periwinkle), *Hibiscus rosa-sinensis* (tropical hibiscus), *Ixora coccinea* (Jungle geranium), and *Lantana camara* (West Indian lantana)*,* while the fruits tested were *Carica papaya* (papaya) and *Mangifera indica* (mango), all of which were locally sourced in Trinidad. These flowers and fruits were selected based on their local availability and abundance in nurseries, typical households and backyards in Trinidad. Flower cuttings were kept in water to maintain freshness; however, parafilm was used to prevent mosquito access to the water source and accidental drowning. The sugar sources from these natural plants were compared against soda combined with RNAi yeast. For these trials 80 mg of Sh.463 yeast was mixed with 200 μL of soda. The mixture was placed in 2 cm x 4 cm sachets made from unstretched Parafilm “M” Laboratory Film (Amcor, Switzerland), and 12 pin sized holes were pierced for mosquito access. Sachets were vertically mounted in the cage, and no additional sugar solution was provided during the experiment.

Experiments were conducted using 15 female and 15 male adult mosquitoes in each 30 cm × 30 cm × 30 cm cage. 4–5 day old adult mosquitoes were starved for 24 h prior to the trials. The mosquito species used included *A. aegypti*, *A. albopictus*, and *C. quinquefasciatus*, all reared from at least the second generation of field-collected specimens gathered locally in ambient conditions at the university insectary. Each set of experiments included three parallel cage trials in which mosquitoes were exposed to sugar as follows:

1. A fruit or flower alone (with survival in these cages demonstrating that the mosquitoes could feed on it, which was required for survival during the six-day trial period).
2. The same fruit or flower as well as a sachet bait station consisting of a paste of Sh.463-Soda (Sh.463 + soda), also referred to as a “Choice.”
3. Sh.463-Soda paste alone, presented as a sachet and serving as a positive control.

The fruits/flowers and baits remained in the cage throughout the duration of the trial without additional sugar supplementation. Humidity was maintained by providing water via a saturated cotton ball in each cage. Each experiment included three biological replicates, with daily mortality recorded over six days. Temperatures ranged from 26.0 °C to 37.0 °C, and relative humidity varied between 34.0% and 90.0% during the experimental periods.

### Assessment of the residual activity of Sh.463-Soda ATSB

The residual activity of the Sh.463 RNAi yeast as an insecticide in soda was evaluated over a fourteen-month period under insectary conditions. RNAi-ATSB mixtures were prepared at a final concentration of 333.3 μg/μL and delivered to mosquitoes in feeders, but with the soda bottle removed and the opening covered. The soda was continuously supplied by adding additional Coca-Cola throughout the experimental period as needed. To prevent mold growth 100g of methyl-p-hydroxybenzoate (Carolina Biological Supply, Burlington, NC) was included per liter of 70% ethanol and added to degassed soda to a final concentration of 0.002%. Control baits (Control + soda) and ASBs (soda with mold inhibitor only) were prepared similarly and included in all experiments.

In an effort to better simulate field conditions, all ATSBs were stored in the insectary for the duration of the experiments, which occurred over the course of 14 months. For each trial, 25 non-blood-fed female mosquitoes were fed overnight with the stored baits, and mortality was recorded daily for six days post-feeding. Fresh soda-mold inhibitor solution was added to replenish the feeder cup whenever drying was observed. The experiment was conducted on four mosquito species: *A. aegypti*, *C. quinquefasciatus* (25 mosquitoes per cage for each), *A. gambiae* and *A. stephensi* (30 mosquitoes per cage for each). At least two biological replicates were performed per species per time point.

### Statistical analyses

Mosquito mortality data across all assays were evaluated as binary outcomes (dead/alive) using generalized linear models (GLMs) and generalized linear mixed models (GLMMs) with a binomial distribution and a logit link function in IBM SPSS Statistics version 31 (Armonk, NY, USA). To account for potential batch effects, inter-trial variation was initially evaluated across all datasets by including trial as a random effect in a GLMM. With the exception of the Trinidad semi-field trials, the GLMM framework was used to evaluate the fixed effects and their interactions. However, the trial variance in the Trinidad semi-field trials proved negligible (<1.0 × 10^-11^). Consequently, the random effect of trial was dropped for the Trinidad semi-field data, justifying the use of a pooled GLM to evaluate the fixed effects of species, sex, floral treatment, cage type, and their interactions. For all experimental data, treatment effects are reported as Estimated Marginal Means (hereafter referred to simply as means) with 95% Wald confidence intervals (CIs), and all pairwise post-hoc comparisons were adjusted using the Bonferroni correction.

## Results

### 1. Efficacy of Sh.463 RNAi yeast treatment across distinct ASB formulations

In laboratory trials, the evaluation of the broad-spectrum insecticidal efficacy of Sh.463 yeast was assessed across five key vector species (*A. aegypti*, *A. albopictus*, *A. gambiae*, *C. pipiens*, and *C. quinquefasciatus*) utilizing three distinct carrier formulations: Soda, Sugar water, and WH. GLMMs were employed for each carrier preparation to evaluate fixed effects of species, treatment, and their interaction, with random effects accounting for trial-level variance.

For the soda-based Sh.463 formulation, a significant main effect of species (*F*_4,120_ = 3.41, *p* = 0.011) was observed, but neither the Sh.463 treatment alone (*F*_2,120_ = 0.002, *p* = 0.998) nor the Species × Sh.463 treatment interaction (*F*_8,120_ = 0.374, *p* = 0.932) reached significance in the overall fixed-effects tests. Pairwise comparisons confirmed highly significant mortality increases in the Sh.463-Soda group relative to both Control-Soda and ASB-Soda across all five species (*p* < 0.001 for all comparisons; Table 1). Observed mortality differences ranged from 93.4% in *A. aegypti* to 98.7% in *C. quinquefasciatu*s, while ASB-soda alone and control yeast treatment mortality differences were non-significant (*p* ≥ 0.182, Fig 1a, Table 1) across all mosquito species, confirming that the soda vehicle alone induces negligible toxicity.

**Table 1:** Pairwise comparison of mean mortality differences of Soda, Sugar and WH RNAi Sh.463 yeast biopesticide formulations across mosquito species.

Species	Comparison	Soda Difference	Soda *p*-value	Sugar Difference	Sugar *p*-value	WH Difference	WH *p*-value
*A. aegypti*	Sh.463 vs. Control	95.1	<0.001	83.4	<0.001	83.4	<0.001
	Sh.463 vs. ASB	93.4	<0.001	82.9	<0.001	82.9	<0.001
	Control vs. ASB	−1.8	0.339	−0.6	0.792	−0.6	0.792
*A. albopictus*	Sh.463 vs. Control	97.3	<0.001	96	<0.001	96	<0.001
	Sh.463 vs. ASB	96	<0.001	94.7	<0.001	94.7	<0.001
	Control vs. ASB	−1.3	0.182	−1.3	0.46	−1.3	0.46
*A. gambiae*	Sh.463 vs. Control	96.5	<0.001	96.4	<0.001	96.4	<0.001
	Sh.463 vs. ASB	94.7	<.0001	98.7	<0.001	98.7	<0.001
	Control vs. ASB	−1.8	0.363	2.2	0.129	2.2	0.129
*C. pipiens*	Sh.463 vs. Control	93.8	<0.001	92.9	<0.001	92.9	<0.001
	Sh.463 vs. ASB	94.2	<0.001	91.1	<0.001	91.1	<0.001
	Control vs. ASB	0.4	0.564	−1.8	0.047	−1.8	0.047
*C. quinquefasciatus*	Sh.463 vs. Control	98.7	<0.001	98.4	<0.001	98.4	<0.001
	Sh.463 vs. ASB	98.7	<0.001	98.4	<0.001	98.4	<0.001
	Control vs. ASB	0	1	0	1	0	1

**Note**: Pairwise comparisons across experimental groups following a GLMM. Adjusted *p*-values through Bonferroni corrections. Control: Control yeast and Sh.463: Sh.463 yeast. ASB = Attractive Sugar Bait; WH = Westham.

**Fig 1:**
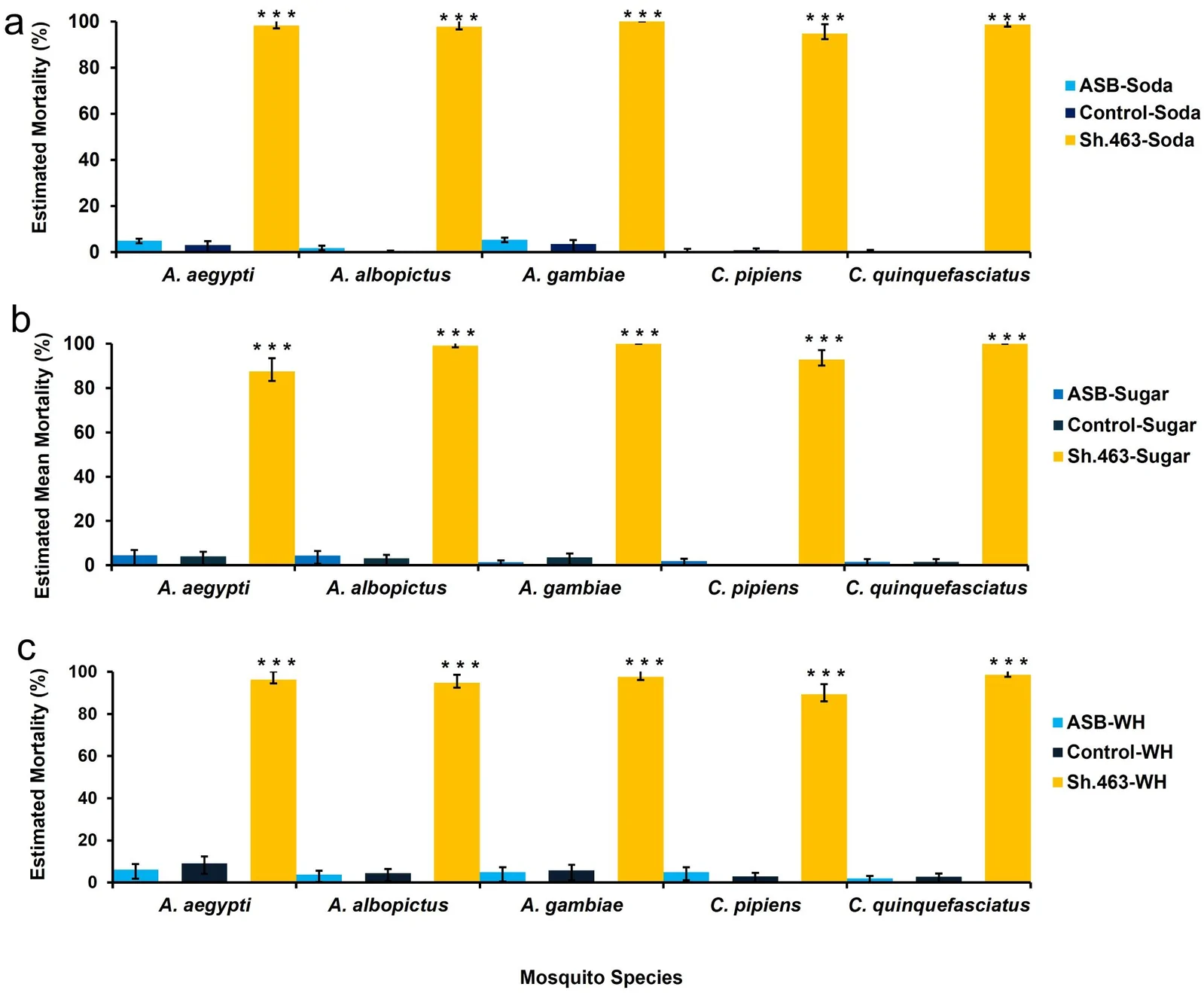
Mortality of various mosquito species exposed to three Sh.463 RNAi yeast treatment formulations. Mean mortality rates (%) for *A. aegypti*, *A. albopictus*, *A. gambiae*, *C. pipiens*, and *C. quinquefasciatus* treated with Sh.463 or control yeast treatments in Soda **(a)**, Sugar **(b)**, and WH **(c)** baits. Error bars indicate 95% CIs. Asterisk *** = *p* < 0.001 between Sh.463 yeast compared with ASB and Control-ASB treatments, which resulted in negligible mortality and did not significantly differ from each other. The number of female mosquitoes tested was N = 225 per species.

The Sh.463-Sugar formulation also induced broad-spectrum mosquito mortality (S1 Table; Fig 1b). While the main fixed effects of Species (*F*_4,102_ = 1.41, *p* = 0.235) and Treatment (ASB, control yeast, or Sh.463 yeast) (*F*_2,102_ = 0.019, *p* = 0.982) were non-significant, a significant Species × Treatment interaction was detected (*F*_8,102_ = 2.16, *p* = 0.037). Pairwise comparisons showed that Sh.463-Sugar induced significantly high mortality across all species (87.5%–100.0%), significantly outperforming Control-Sugar and ASB-Sugar controls (*p* < 0.001 for all comparisons; Table 1). Background mortality in control groups remained low (0.9%–4.6%, Fig 1b, S2 Table) with no significant difference between control types across most species (*p* > 0.05), except for a minor variation in *C. pipiens* (*p* = 0.047; Table 1). The random effect associated with experimental trial was negligible (variance = 0.0032, *p* = 0.929), indicating high reproducibility across replicates.

Testing of the WH-based yeast formulation revealed significant main effects for both Species (*F*_4,111_ = 3.40, *p* = 0.012) and Treatment (F _2,111_ = 439.26, *p* < 0.001), along with a significant Species × Treatment interaction (*F*_8,111_ = 2.29, *p* = 0.026). Consistent with the other sugar carriers, pairwise comparisons demonstrated that the Sh.463-WH formulation induced significantly higher mortality compared to both Control-WH and WH-ASB controls across all species (*p* < 0.001). Mortality differences relative to controls ranged from 89.4% in *C. pipiens* to 98.6% in *C. quinquefasciatus* (S1 Table; Fig 1c). Control comparisons yielded minimal, non-significant differences for nearly all species (*p* > 0.05), with only *C. pipiens* exhibiting a minor divergence (-1.8%, *p* = 0.047; Table 1). The corresponding negative ASB control data are shown in (S2 Table) for ASB-Soda, ASB-Sugar and ASB-WH.

In summary, across all three delivery carriers (Soda, Sugar water, and WH), the lethal Sh.463 RNAi yeast biopesticide consistently produced near complete or complete mortality across diverse mosquito genera (*Aedes, Anopheles,* and *Culex*). In all cases, the control groups yielded minimal background mortality, compared to the lethal RNAi yeast treatments (Tables 1, S1). Together, these results establish that the yeast biopesticide platform maintains uniform, high-potency insecticidal activity regardless of formulation type, supporting its versatility for field implementation against multiple mosquito disease vectors.

### 2. Soda-based yeast biopesticides outperform alternative sugar carriers in dual-choice assays to drive mosquito mortality across multiple vector species

Dual-choice assays were performed to assess the most effective sugar bait carrier (ASB-Sugar, ASB-Soda and ASB-WH). Overall, the experimental baiting system demonstrated exceptional stability, with negligible variation observed between independent experimental trials (*variance* = 0.008, *p* = 0.494). The Type III tests of fixed effects demonstrated that adult mosquito mortality was strongly influenced by the choice of Sh.463 yeast carrier (*F*_*2*,103_ = 441.063, *p* < 0.001) and varied significantly across the tested mosquito species (*F*_4,103_ = 19.736, *p* < 0.001) (Table 2). Notably, a highly significant interaction was detected between species and Sh.463 yeast carrier (*F*_8,103_ = 8.061, *p* < 0.001), confirming that the biological efficacy or deterrent properties of individual carrier formulations are highly species-specific. In contrast, the choice of bait pair had no significant impact on mortality outcomes (*F*_1,103_ = 1.019, *p* = 0.315), validating that the choice of sugar carrier was the primary driver of yeast bait performance rather than the specific pairing.

**Table 2:** Effects of species, bait pairing and Sh.463 yeast carrier on mosquito mortality.

Fixed Effect	F	df1	df2	*p*-value
Mosquito Species	19.736	4	103	<0.001
Bait Pair	1.019	1	103	0.315
Sh.463 yeast carrier	441.063	2	103	<0.001
Mosquito Species * Sh.463 yeast carrier	8.061	8	103	<0.001

**Note**: Abbreviations: df1, numerator degrees of freedom; df2, denominator degrees of freedom.

Bait Pair: Bait Pair 1 = Soda-Treatment vs. WH-Control and Soda-Control vs. WH-Treatment; Bait Pair 2 = Soda-Treatment vs. Sugar-Control and Soda-Control vs. Sugar-Treatment.

These trials revealed that the soda-based Sh.463 yeast formulation consistently achieved the highest predicted mean mortality rates across all five mosquito species in the choice-test setups, resulting in 81.4% to 95.3% mortality rates, and reaching a peak efficacy of 95.3% mortality in *C. pipiens* (Fig 2, S3 Table; corresponding negative ASB control data are shown in S4 Table). The results in *C. pipiens* were further confirmed using fluorescent dye marked dual-choice assays which verified that soda was the preferred carrier for delivering lethal yeast, driving significantly higher ingestion rates than WH (*χ*^2^ = 162.39 and *χ*^2^ = 34.59, respectively; *p* < 0.001; S5 and S6 Tables).

**Fig 2:**
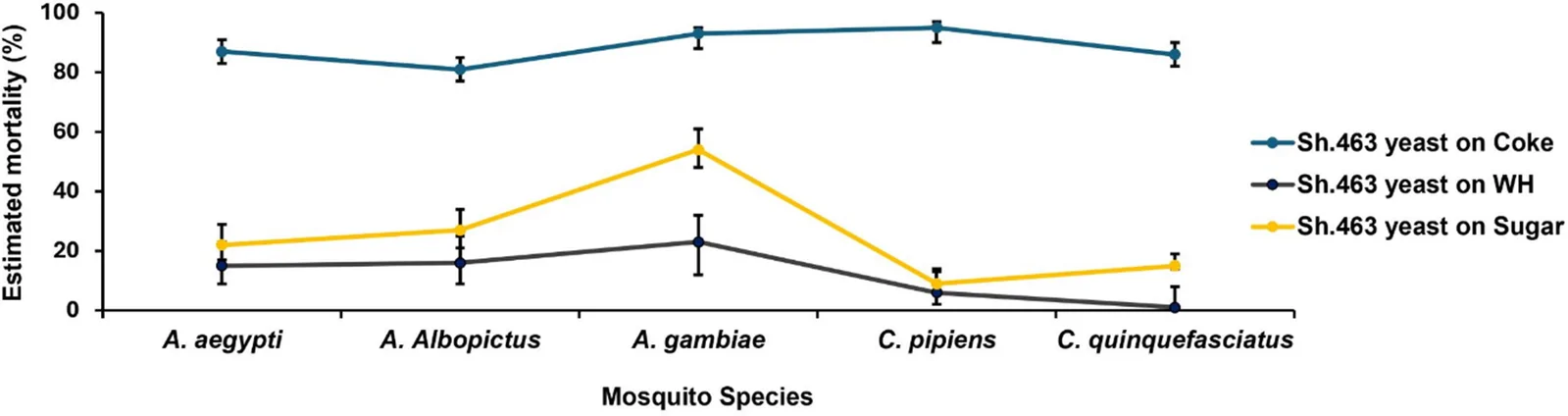
Mean adult mosquito mortality rates across five mosquito species exposed to Sh.463 yeast delivered using soda, WH, or sugar water in dual-choice bioassays. Error bars represent 95% CIs. Pairwise comparisons of mortality rate differences between soda, sugar and WH yeast formulations are described in Table 3. The total number of females was N = 150 per group.

In marked contrast, the alternative WH and sugar water yeast baits induced significantly lower mean mortalities in dual-choice assays in which sugar- or soda-based baits were provided as alternatives, remaining below 30% for most species except for the sugar water-based Sh.463 yeast choice in *A. gambiae*, which reached 54.0% (Fig 2, S3 Table; corresponding negative ASB control data are shown in S7 Table). These results exhibit the substantial performance advantage of utilizing Soda as the sugar bait carrier which drove high Sh.463-induced lethality even when presented alongside a competing matrix choice.

In summary, the dual-choice experiments further confirmed that soda is the most effective sugar bait carrier for the Sh.463 yeast biopesticide across all five tested species (*p* < 0.001 for all comparisons, Table 3). In every species, the mortality rate induced by the soda-based yeast ATSB was significantly higher than both the WH-based yeast ATSB and the sugar water-based yeast ATSB (Table 3). Pairwise choice comparisons explicitly confirmed this advantage, showing that the soda carrier drove an absolute increase in predicted mortality of 39.0% to 89.1% compared to alternative matrices across all species tested (Table 3). Every individual comparison against soda was highly significant (*p* < 0.001), with the most pronounced differences observed in *C. pipiens*, in which soda outperformed WH by 89.1% and sugar water by 85.9% (Table 3). Furthermore, the tight 95% CIs across all carrier groups underscore the high precision and reliability of these model estimates (Fig 2, Tables 3 and S3).

**Table 3:** Pairwise comparisons of model-estimated adult mosquito mortality rates between the soda carrier and alternative delivery matrices within each species.

Species	Comparison (Vs Soda)	Mean Difference	95% CI	*p*-value
*A. aegypti*	Soda vs. WH	0.725	(0.64, 0.81)	<0.001
	Soda vs. Sugar	0.653	(0.56, 0.75)	<0.001
*A. albopictus*	Soda vs. WH	0.658	(0.57, 0.75)	<0.001
	Soda vs. Sugar	0.545	(0.44, 0.65)	<0.001
*A. gambiae*	Soda vs. WH	0.702	(0.61, 0.79)	<0.001
	Soda vs. Sugar	0.39	(0.28, 0.50)	<0.001
*C. pipiens*	Soda vs. WH	0.891	(0.83, 0.95)	<0.001
	Soda vs. Sugar	0.859	(0.79, 0.92)	<0.001
*C. quinquefasciatus*	Soda vs. WH	0.851	(0.80, 0.91)	<0.001
	Soda vs. Sugar	0.714	(0.63, 0.80)	<0.001

**Note:** Mean differences represent back-transformed mortality proportions between the soda yeast formulation and alternative carrier matrices. 95% CIs and *p*-values are derived from pairwise comparisons evaluated within a GLMM (α = 0.05). The total number of females was N = 150 per group.

The likelihood of mortality was highly dependent on the choice of sugar carrier, with GLMM odds ratios (OR) quantifying the magnitude of these effects. Notably, soda was most preferred as the sugar bait carrier option for the Sh.463 yeast, demonstrating a very highly significant overall increase in lethal efficacy as an ATSB compared to the sugar water-based ATSB (OR = 35.86 [95% CI: 19.80–64.95], *p* < .001) (Table 4). Meanwhile, WH was less preferred as a sugar carrier option, yielding a significantly lower OR of 0.07 [95% CI: 0.02–0.32], *p* < .001, indicating a reduced feeding preference that limits its performance compared to sugar water-based yeast bait.

**Table 4:** Estimated odds ratios for mosquito mortality across species, bait pairings and Sh.463 yeast carriers.

Model Term	Odds Ratio (OR)	95% CI	*p*-value
**Mosquito Species**			
*A. aegypti*	1.601	(0.87, 2.96)	0.132
*A. albopictus*	2.085	(1.15, 3.79)	0.016
*A. gambiae*	6.65	(3.78, 11.71)	<0.001
*C. pipiens*	0.583	(0.28, 1.22)	0.152
*C. quinquefasciatus*	1	Reference	–
**Bait Pair**			
Bait Pair 1	0.166	(0.86, 1.63)	0.315
Bait Pair 2	1	Reference	–
**Sh.463 Yeast Carrier**			
Soda	35.864	(19.80, 64.95)	<0.001
WH	0.07	(0.02, 0.32)	<0.001
Sugar water	1	Reference	–
**Key interactions**			
Species* Sh.463 Yeast Carrier			
*A. aegypti ×* Soda	0.682	(0.31, 1.87)	0.333
*A. aegypti ×* WH	8.81	(1.78, 43.72)	0.008
*A. albopictus ×* Soda	0.331	(0.16, 34.94)	0.004
*A. albopictus ×* WH	7.102	(1.44, 34.94)	0.016
*A. gambiae ×* Soda	0.316	(0.14, 0.69)	0.004
*A. gambiae ×* WH	3.573	(0.75, 17.03)	0.109
*C. pipiens ×* Soda	5.437	(2.02, 14.62)	<0.001
*C. pipiens ×* WH	9.07	(1.62, 50.73)	0.013

**Note**: Odds Ratios (ORs) and 95% CIs are relative to the reference categories: Mosquito species (*C. quinquefasciatus)*, Bait Pair 2 (Bait Pair), and Sh.463 yeast carrier (Sugar Water). WH: Westham bait. An OR > 1 indicates higher odds of mortality compared to the baseline, while an OR < 1 indicates lower odds. All pairwise comparisons were adjusted using a Bonferroni correction with significance level set at *p* < 0.05.

The significant interaction terms further highlight species-specific responses. For instance, the *C. pipiens* × WH interaction revealed strong synergistic efficacy (OR = 9.07 [95% CI: 1.62–50.73, *p* = 0.013]), confirming that this combination is highly effective for *C. pipiens*. In contrast, the *A. albopictus* × Soda interaction (OR = 0.33 [95% CI: 0.16–0.70, *p* = 0.004]) demonstrated an antagonistic effect, suggesting that even though soda was the most preferred delivery option overall, it was less effective for this specific species (Table 4).

### 3. Evaluation of RNAi yeast-soda ATSB performance in semi-field trials conducted in the presence of competing natural sugar sources in Trinidad

To further assess the performance of the Sh.463 RNAi yeast pesticide in soda in a competitive tropical semi-field setting in Trinidad, the impact of the competing natural sugar sources was evaluated by placing a flower or a piece of fruit in the Sh.463-Soda mosquito cages. The semi-field trials were conducted across three target mosquito species (*A. aegypti*, *A. albopictus*, and *C. quinquefasciatus*) exposed to diverse local flora in both no-choice (Sh.463-Soda ATSB alone) and choice (Sh.463-Soda + competing fruit for flower) environments. The mosquito strains used in these studies were generated from locally collected field specimens.

GLM modeling revealed that mosquito mortality was mainly driven by the experimental feeding regime and floral and fruit background, as well as their higher-order interactions with species and sex (Table 5). The corresponding negative control for flowers and fruits only, for three mosquito species are shown in S8 Table. Highly significant higher-order interactive terms including Species × Floral treatment (*χ*^2^ = 3993.58, *df* = 11, *p* < 0.001) and Floral treatment × Cage Type (*χ*^2^ = 92.52, *df* = 5, *p* < 0.001) confirmed that lethal yeast efficacy and competitive floral distraction are strongly modulated by distinct species-specific and sex-specific behavioral dynamics (Table 5), though global baseline main effects for species (*χ*^2^ = 0.20, *df* = 2, *p* = 0.905) and sex (*χ*^2^ = 2.00, *df* = 1, *p* = 0.158) were not independently significant on their own.

**Table 5:** Factors influencing toxic yeast bait efficacy in the presence of competitive floral backgrounds.

Source of Variation	Wald *χ*^2^	*df*	*p*-value
Mosquito Species	0.20	2	0.905
Sex	2.00	1	0.158
Floral Treatment	1693.23	6	<0.001
Cage Type	1879.13	1	<0.001
Species × Sex	2.132	2	0.344
Species × Floral-fruit Treatment	3993.58	11	<0.001
Species × Cage Type	49.17	2	<0.001
Sex × Floral-fruit Treatment	49.74	6	<0.001
Sex × Cage Type	3.86	1	0.049
Floral-fruit Treatment × Cage Type	92.52	5	<0.001

**Note**: Wald *χ*^2^ tests of fixed effects and interactions derived from GLM.

Statistical significance indicated at *p* < 0.05 adjusted through Bonferroni correction.

Cage Type represents Choice = Flower/fruit +Sh.463 yeast only (Cage 1) and Sh.463 yeast only (Cage 2) feeding regimes.

Mosquito mortality was significantly influenced by baseline mosquito sex, floral treatments, and the experimental feeding regime. Broadly, *A. aegypti* exhibited significantly higher overall baseline mortality compared to *C. quinquefasciatus* (OR = 2.36, 95% CI [1.11-4.82], *p* = 0.019), while males demonstrated a pronounced survival advantage over females across all experimental treatments (OR = 0.43, 95% CI [0.24-0.78], *p* = 0.005) (Table 6). The critical role of alternative sugar source competition was clearly established by the strong main effect of the Sh.463-Soda experimental feeding regime, with the presence of competing sugar source configurations (Cage type, Table 6) driving significant global reduction in Sh.463 yeast efficacy (OR = 0.19, 95% CI [0.11-0.35], *p* < 0.001), meaning that the presence of competing flowers or fruits significantly reduces overall mortality and yeast efficacy. However, the degree of this competitive deterrence was highly dependent on the specific floral-fruit background.

**Table 6:** Effects of natural sugar sources on the efficacy of the Sh.463-Soda ATSB.

Model Term/ Parameter	Odds Ratio (OR)	95% CI	*p*-value
**Mosquito Species**			
*A. aegypti*	2.36	(1.11, 4.82)	0.019
*A. albopictus*	0.8	(0.42, 1.54)	0.508
*C. quinquefasciatus*	1	(Reference)	—
**Sex**			
Male	0.43	(0.24, 0.78)	0.005
Female	1	(Reference)	—
**Cage Type**			
Choice	0.19	(0.11, 0.35)	<0.001
Sh.463 Yeast Only	1	(Reference)	—
**Floral-fruit Treatment**			
*A. cathartica*	5.12	(2.43, 10.80)	<0.001
*C. roseus*	0.70	(0.45, 1.10)	0.121
*H. rosa-sinensis*	42.33	(6.38, 280.88)	<0.001
*I. coccinea*	2.44	(1.36, 4.36)	0.003
*L. camara*	1.74	(1.02, 2.96)	0.042
*C. papaya*	2.9	(1.57, 5.36)	<0.001
*M. indica* (Baseline)	1	(Reference)	—
**Floral-fruit** **Treatment × Choice**			
*A. cathartica ×* Choice	12.7	(5.24, 30.80)	<0.001
*C. roseus ×* Choice	>100	N/A	<0.002
*H. rosa-sinensis ×* Choice	13.36	(6.87, 26.02)	<0.001
*I. coccinea ×* Choice	1.83	(0.84, 4.01)	0.131
*L. camara ×* Choice	1.79	(0.91, 3.51)	0.09
*C. papaya ×* Choice	0.47	(0.22, 1.00)	0.05
Sh.463 Yeast Only	1	(Reference)	—

**Note**: Odds Ratios (ORs) and 95% CIs are relative to the reference categories: *C. quinquefasciatus*, Females, Sh.463 yeast only, and *M. indica*. An OR > 1 indicates higher odds of mortality compared to the baseline, while an OR < 1 indicates lower odds of mortality. All pairwise comparisons were adjusted using a Bonferroni correction with a *p* < 0.05 significance level.

The presence of certain floral-fruit backgrounds inherently drove extreme baseline lethality, most notably *H. rosa-sinensis* (OR = 42.33, 95% CI [6.38-280.88], *p* < 0.001) and *A. cathartica* (OR=5.12, 95% CI [2.43-10.80], *p* < 0.001), and their corresponding interactive terms with Choice environments (OR = 13.36 and OR =12.70, respectively; both *p* < 0.001) confirmed a significant failure to outcompete the lethal Sh.463-Soda yeast formulation (Table 6). A failure to deter feeding was most extreme in the presence of *C. roseus*, which yielded an exceptionally high interactive odds ratio (OR > 100, *p* < 0.002), indicating that the presence of *C. roseus* offered virtually no competitive distraction away from the lethal yeast. Conversely, *C. papaya* emerged as a highly effective competitive deterrent, significantly suppressing expected mortality margins (OR = 0.47, 95% CI [0.22-1.00], *p* = 0.05) (Table 6).

Pairwise post-hoc comparisons further clarified how the presence of alternative floral-fruit resources compromised the efficacy of the toxic yeast bait across species and sexes (Table 7). For *A. aegypti*, the introduction of floral-fruit resources significantly reduced bait efficacy across the majority of floral-fruit backgrounds, as visually demonstrated by the overall downward movement of the choice trendlines relative to the Sh.463-Soda only trendline; mortality rates were very significantly reduced in the presence of *C. papaya*, dropping from 92% to 18% in females and from 97% to 28% in males (both *p* < 0.001; Fig 3a, S9 Table). Interestingly, within these choice environments, *A. aegypti* exhibited significant sex-specific mortality differences under specific floral-fruit backgrounds (S9 Table); females experienced significantly higher mortality than males in *C. roseus* (88% vs. 78%, *p* = 0.031) and *L. camara* (59% vs. 50%, *p* = 0.031) choice backgrounds, whereas males were significantly more vulnerable than females in *H. rosa-sinensis* choice cages (84% vs. 70%, *p* = 0.021).

**Table 7:** Sex-differentiated mortality comparisons within species across Sh.463-Soda only and floral-fruit background feeding regimes.

Species/ Floral-fruit Background	% Female Mortality		% Male Mortality			
	Choice	Sh.463 yeast Only	Choice	Sh.463 yeast Only	Sex Diff*. p*-value (Choice)	Sex Diff. *p*-value (Sh.463 yeast Only)
***A. aegypti***						
*A. cathartica*	79	100	95	100	1	N/A
*C. roseus*	88	93	78	92	0.031	0.036
*H. rosa-sinensis*	70	100	84	100	0.021	N/A
*I. coccinea*	68	97	62	97	0.663	N/A
*L. camara*	59	95	50	96	0.031	0.312
*C. papaya*	18	92	28	97	<0.001	0.501
*M. indica*	40	94	21	91	0.505	0.501
***A. albopictus***						
*A. cathartica*	48	85	81	98	<0.001	0.002
*C. roseus*	96	86	92	81	0.163	0.777
*H. rosa-sinensis*	93	100	96	100	0.645	N/A
*I. coccinea*	76	84	70	86	0.633	0.56
*L. camara*	86	91	80	91	0.788	0.396
*C. papaya*	77	96	85	98	0.267	0.303
*M. indica*	66	85	41	77	<0.001	< 0.001
***C. quinquefasciatus***						
*A. cathartica*	100	100	100	100	N/A	N/A
*C. roseus*	88	73	71	60	<0.001	0.271
*H. rosa-sinensis*	100	100	100	100	N/A	N/A
*I. coccinea*	98	99	97	99	N/A	0.073
*L. camara*	72	88	55	85	0.167	0.174
*C. papaya*	47	91	52	95	0.391	N/A
*M. indica*	58	88	27	76	0.047	0.366

**Note:** GLM generated values reflect estimated mean mortality percentages under Choice (Flower/fruit + Sh.463 Yeast) and Sh.463 yeast only feeding regimes. N/A, not applicable indicates comparisons where variance could not be calculated due to 100% mortality in both comparison groups. Choice and Sh.463 yeast only *p-values* report adjusted significance levels for post-hoc pairwise comparisons between sexes (females and males) within Choice and Sh.463 yeast only setups, respectively. Tests were conducted on male (n = 15) and female (n = 15) mosquitoes for three replicate trials (N = 45).

**Fig 3:**
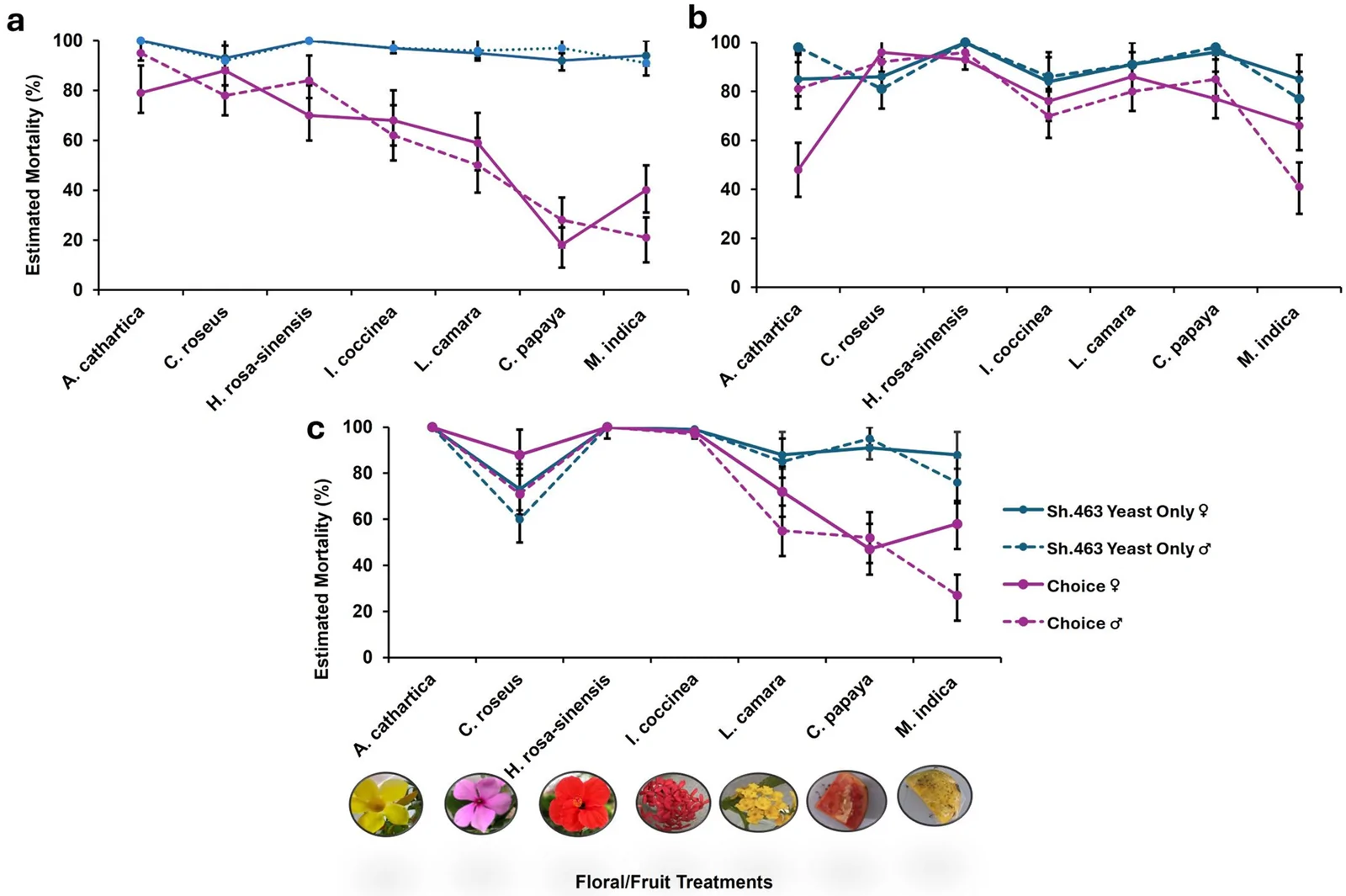
Sh.463 yeast-induced mosquito mortality in the presence of competing natural sugar sources. The mean mortality of *A. aegypti* **(a)**, *A. albopictus* **(b)**, and *C. quinquefasciatus* **(c)** across floral-fruit treatments are shown**.** Sex-specific mean mosquito mortality in choice (Floral-fruit background + Sh.463-Soda) and Sh.463-Soda alone assays. Error bars indicate 95% CIs. Pairwise comparisons between Choice and Sh.463 only assays across mosquito species by gender are presented in Tables 7 and S9. The results were compiled from three replicate cage trials, each containing 15 male and 15 female mosquitoes presented with Sh.463-Soda sachets alone or in the presence of various fruits or flowers.

Conversely, *A. albopictus* exhibited a largely unchanged feeding profile with tightly overlapping Sh.463-Soda and choice trendlines across most floral-fruit contexts, with the exception of a severe mortality reduction in females exposed to *A. cathartica* (dropping from 85% to 48%, *p* < 0.001; Fig 3b, S9 Table) and moderate declines in *C. papaya* and *M. indica* environments. This *A. albopictus* drop in mortality in the presence of *A. cathartica* highlights a pronounced sex-specific difference, as males maintained significantly higher mortality than females in both choice (81% vs. 48%, *p* < 0.001) and Sh.463 yeast configurations (98% vs. 85%, *p* = 0.002; S9 Table), revealing that female feeding behavior is much more heavily distracted by *A. cathartica* flowers than male behavior.

For *C. quinquefasciatus*, the impact of competitive floral-fruit interference was remarkably minimal across various floral-fruit backgrounds, for example *A. cathartica* and *H. rosa-sinensis*, in which 100% mortality was maintained (*p* = 1.000; Fig 3c, S9 Table). However, *C. quinquefasciatus* mortality was heavily compromised in the presence of *C. papaya* (dropping from 91% to 47% in females and from 95% to 52% in males; *p* < 0.001) or *M. indica* (dropping from 88% to 58% in females and from 76% to 27% in males; *p <* 0.001). These results reveal that while *C. quinquefasciatus* and *A. albopictus* are generally more resistant to natural floral-fruit distractions than are *A. aegypti*, specific competitive fruit and floral-fruit matrices still significantly undermine Sh.463-Soda bait performance. Furthermore, significant sex-specific differences emerged in *C. quinquefasciatus*, with females exhibiting higher mortality than males, notably under *C. roseus* choice (88% vs. 71%, *p* < 0.001) and *M. indica* choice (58% vs. 27%, *p* = 0.047; Table 7) configurations, indicating that alternative floral-fruit contexts may be a stronger competitive deterrence to *C. quinquefasciatus* males than to females. Overall, the presence of alternative floral resources significantly altered yeast bait performance. The competitiveness of the lethal yeast biopesticide in choice environments was highly dependent on the specific floral background, with the degree of competitive deterrence being heavily modulated by mosquito species, sex, and floral resource.

### 4. Semi-field evaluation of a soda bottle feeder for Sh.463-Soda ATSBs

A prototype soda bottle feeder was assessed in semi-field outdoor conditions in South Bend, IN. These trials were conducted on a *C. pipiens* strain established from a local collection. Mosquitoes were observed landing on and feeding from the soda feeders containing ASB-Soda, Control-Soda or the Sh.463-Soda ATSB. Mosquito mortality was significantly higher for the Sh.463-Soda treatment (94.7%, 95% CI: [0.902-0.97]) compared to both ASB-Soda alone (10.6%, 95% CI: [0.07- 0.17]; *p* < 0.001) or Control -Soda (8.8%, 95% CI: [0.053-0.144]; *p* < 0.001). Mortality did not differ significantly between the ASB-Soda and Control-Soda negative control groups (*p* = 0.532), with baseline control mortality remaining negligible despite the high variability of temperature during the study period; the low number of control deaths demonstrated that the mosquitoes consuming the Control-Soda and ASB-Soda were able to tolerate these conditions and survive while drinking and eating from the feeders and that mortality was primarily being driven by the Sh.463 yeast treatment (Fig 4).

**Fig 4:**
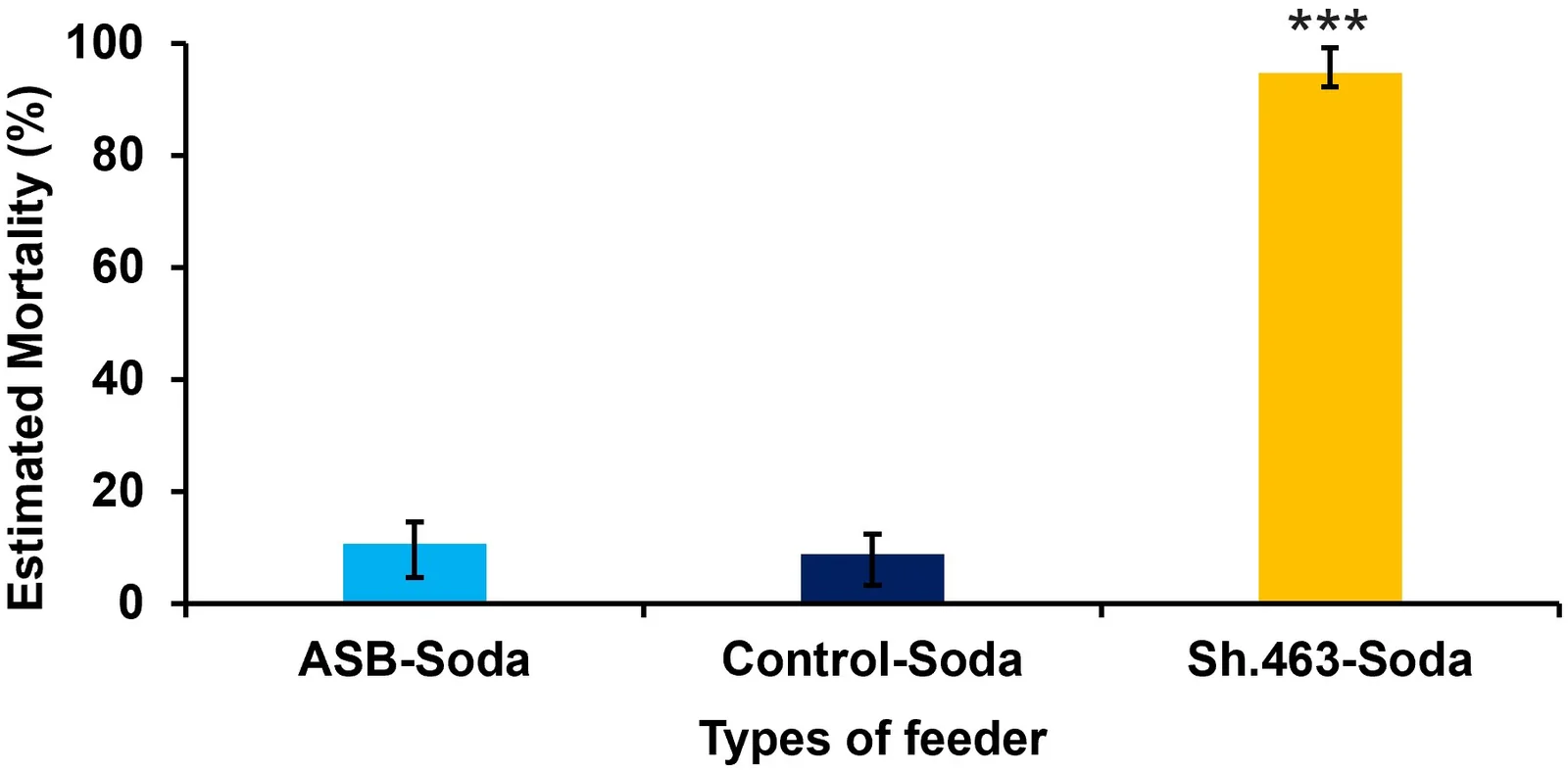
Semi-field evaluation of a soda bottle feeder for delivery of the Sh.463-Soda ATSB Mean mosquito mortality with error bars indicating 95% CIs from a binomial GLMM. Asterisks denote a statistically significant difference compared to ASB-Soda or Control-Soda (*** p < 0.001). Results were compiled from three biological trials, each with three replicate cages per treatment group, N = 225.

### 5. Pronounced residual activity of Sh.463-Soda over 14 months

Residual activity of the Sh.463-Soda bait was evaluated in laboratory trials conducted over the course of 14 months. The Sh.463-Soda ATSB and Control-Soda bait were stored in the insectary to simulate field conditions for the duration of the experimental period. Throughout the trial, and across four different mosquito species evaluated, the Sh.463-Soda retained high insecticidal activity in ATSB trials conducted intermittently on *A. aegypti, A. gambiae, A. stephensi* and *C. quinquefasciatus* throughout the trial period (Fig 5)*.* The Sh.463-Soda ATSB demonstrated exceptional long-term stability and near-complete residual insecticidal activity across four major mosquito species (*A. aegypti, A. gambiae, A. stephensi,* and *C. quinquefasciatus)* in ATSB lab trials (Fig 5, S10 Table).

**Fig 5:**
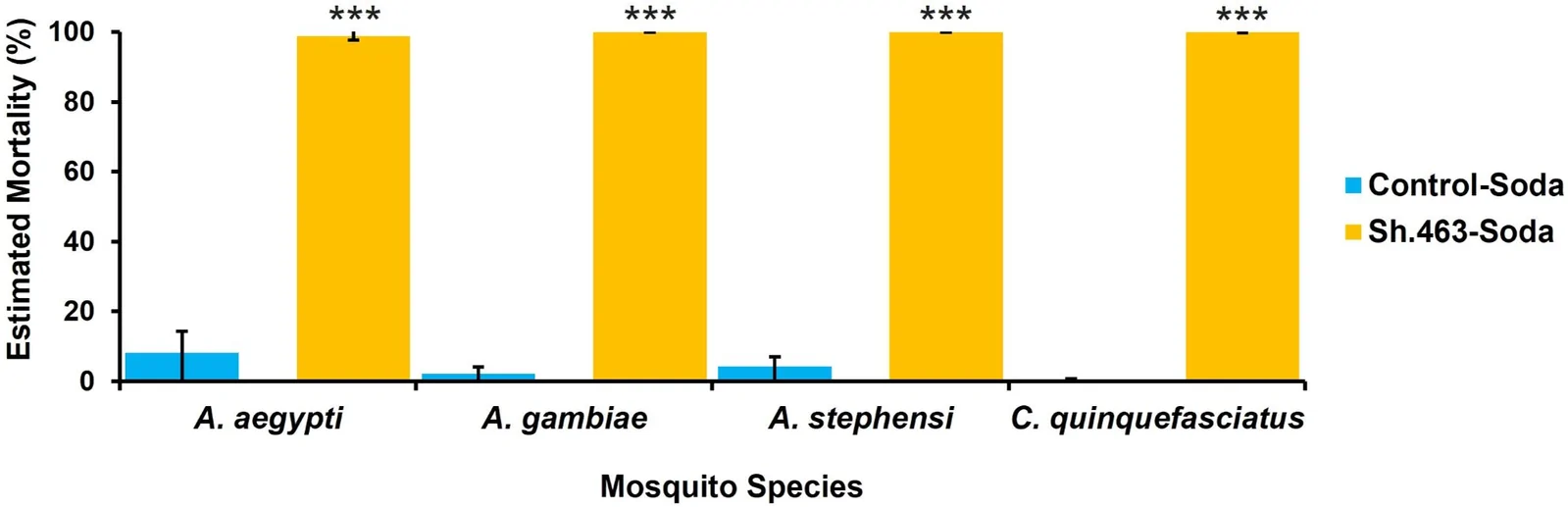
14-month residual activity of the Sh.463-Soda ATSB. The residual insecticidal activity of Sh.463-Soda across four mosquito species evaluated across 14 months is shown. Mean mortality rates documented for *A. aegypti, A. gambiae, A. stephensi* and *C. quinquefasciatus* (n = 2 – 6 trials) following exposure to the Sh.463-Soda and Control-Soda formulations in soda bottle feeders. Bars represent estimated mean mortality rates. Error bars indicate 95% CIs. *** denotes significant differences (*p* < 0.001) in mortality between the Sh.463-Soda and control groups generated using GLMM.

*C. quinquefasciatus* displayed 100% mortality during months 2–14 (S10 Table). Similarly, in *A. aegypti*, complete mortality (100%) was recorded at months 4 and 13, and a mortality of 93.0 ± 4.12% was recorded at month 10. Moreover, the Control-Soda groups consistently showed minimal mortality (0–10%). *A. gambiae* was evaluated at months 10 and 14, and the species exhibited a high susceptibility, with a mortality of 100% observed in the Sh.463-Soda groups and Control-Soda mortality rates of 0.7 ± 0.67% and 4.5 ± 1.50%, respectively (S11 Table). *A. stephensi* also showed high susceptibility during the five timepoints it was tested with the bait. Mortality in the Sh.463-Soda group ranged from 99.3 ± 1.50% in month 1–100% in subsequent months, while mortality in the Control-Soda group remained low (0.0 ± 0.0% – 6.0 ± 2.00%) (S11 Table).

A random effect analysis showed group-level baseline variation (*variance* = 2.443), which was not statistically significant (*p* = 0.113). In the fixed effects analysis, while neither main effect (Species: *p* = 0.996; Sh.463 yeast: *p* = 0.802) nor the control covariate (Month: *p* = 0.868) was significant on its own, there was a statistically significant interaction between species and treatment (*F*_3,103_ = 6.213, *p* = 0.001; S12 Table). Overall, the model was highly significant (*p* < 0.001), indicating that while baseline group variation was modest and main factors showed no broad effect across groups, treatment performance differed substantially depending on the specific species evaluated. Throughout the study, monthly mean mortality rates in the Sh.463-Soda groups remained consistently high between 98.8%-100.0% (95% CI: [93.4-100.0] (Fig 5 and S10 Table), whereas Control-Soda mortality remained exceptionally low at 0% to 14.0%, yielding statistically significant pairwise mortality differences between Sh.463 and control for every species evaluated (*p* < 0.001; Fig 5). Statistically, the lack of significant variation from the calendar month covariate (*p* = 0.868) confirms an absence of inter-trial temporal variation, which is indicative of a durable, uniform Sh.463-Soda efficacy without seasonal degradation over extended deployment periods.

## Discussion

Maximizing the effectiveness of ATSBs as a scalable and environmentally sustainable vector control strategy requires optimizing their capacity to induce feeding in target mosquito populations [33]. This depends on the effective integration of both the attractant and sugar components, which work together to lure mosquitoes to the baits, drawing them away from natural sugar sources, and ensuring ingestion of the toxic insecticide during sugar feeding [41]. Enhancing ATSB delivery mechanisms not only improves kill rates but also extends coverage to a wider range of ecological settings and mosquito species [42].

Building on the importance of optimizing ATSB technology for mosquito control [42,43], we showed that the active ingredient Sh.463 yeast delivered in three sugar types: Coca-Cola (Soda), 10% sucrose (Sugar), and WH, achieved high mortality across five mosquito species: *A. aegypti*, *A. albopictus*, *A. gambiae*, *C. pipiens*, and *C. quinquefasciatus* (Fig 1, S1 Table). Mortality rates in the RNAi treatment groups consistently exceeded 75%, while mortality in the control yeast and ASB-only groups were negligible. These findings highlight the capacity of this yeast active ingredient to perform effectively across different sugar bait types, underscoring its versatility and operational flexibility for field applications [9,11,44]. We have previously shown that Sh.463 and other RNAi-based mosquito-targeted pesticides can effectively induce species-specific mortality, thus reducing environmental risks [8,9,11,44,45]. The oral delivery mechanism, specificity of the ATSB to target organisms, and scalable production of the yeast make this system well-suited for deployment in diverse ecological settings, particularly in areas where conventional interventions are failing due to resistance and behavioral avoidance [46].

Among the sugar formulations tested, soda emerged as the most effective vehicle for RNAi yeast delivery (Fig 2, S3 Table). We observed consistently strong mortality rates exceeding 93% across all species and reaching 100% for several Sh.463-Soda trials. Beyond the intrinsic insecticidal activities of RNAi yeasts [8,9,11,44,45], components of soda (Coca-Cola) such as high sugar content, carbonation byproducts, or flavorings, may enhance phagostimulatory cues and palatability, thereby increasing mosquito feeding rates [35,41,47]. The ability of the Sh.463-Soda formulation to effectively kill mosquitoes in the presence of several competing natural sugar sources under semi-field conditions (Fig 3) suggests that it is a promising ATSB that warrants further investigation in broader settings. In addition to examining the field efficacy of Sh.463-Soda ATSBs for mosquito control, it will also be important to assess what non-target organisms might feed from the yeast-soda feeders and to confirm that the yeast, which was designed to be specific to mosquitoes, is not toxic to non-target species.

Previous studies have shown that incorporating fruit juices or fermenting sugars into ATSBs increases attractiveness and efficacy in *Anopheles* and *Aedes* mosquitoes [35,48]. Importantly, the increased mortality observed with lethal RNAi yeast + soda formulations in our study likely correlated with higher ingestion and delivery of RNAi-yeast load, as further illustrated by dye-marked dual-choice assays (S5 and S6 Tables). These results support our hypothesis that combining RNAi yeast with a highly attractive sugar source can significantly enhance its entomological efficacy and operational viability as an ATSB-based mosquito control tool. Future studies should investigate the mechanisms underlying soda’s enhanced attraction, as well as explore its field-based validation.

Additionally, the potential synergistic effects between RNAi yeast insecticides and soda-based sugar formulations warrant further exploration to optimize their utility as ATSB tools. The efficacy of the RNAi yeast + soda ATSB in malaria vector species, *A. gambiae* and *A. stephensi,* demonstrated here and in a recent study [49] are also noteworthy, as malaria vectors are increasingly resistant to conventional control methods [3]. Reports from recent large-scale ATSB field studies [29,50], which tested ATSB technologies against *A. gambiae,* highlight the need for a more effective, scalable, user-friendly and environmentally sustainable ATSB tool. The RNAi yeast-soda bait evaluated in our study presents a promising platform that could potentially meet these criteria and advance ATSB-based mosquito control strategies pending further investigations in the field. For example, given that the WH bait-based ATSBs failed to reduce the incidence of malaria in recent trials that examined the epidemiological impacts of this intervention [29], it would be useful to examine the attractiveness of the RNAi-yeast based soda baits to *Anopheles spp*. in semi-field trials conducted in the presence of natural sugar source competitors. Such trials, as well as examination of *Anopheles spp.* mosquito feeding rates on Sh.463-Soda in the field, would permit further examination of mosquito attractiveness to these baits. The semi-field trials presented herein (Fig 3, S9 Table) suggest that placing RNAi yeast-soda ATSB feeders near human dwellings requiring mosquito control could result in mitigating mosquito densities alongside the use of other mosquito control interventions pending further field evaluations of the Sh.463-Soda ATSB. This is particularly important for *A. aegypti,* which was impacted by a variety of different fruits and flowers (Fig 3a). The observed impacts of *L. camara* flowers (Fig 3a, S9 Table) is consistent with observations made in the study by Upshur and colleagues [35] in which this flower was believed to be attractive to mosquitoes as a plant-sugar source, likely due to the presence of volatile benzaldehydes.

In terms of fruits, we observed all three mosquito species feeding on both *C. papaya* and *M. indica*, and the impacts of these fruits on RNAi yeast + soda efficacy in *A. aegypti, A. albopictus,* and *C. quinquefasciatus* are of potential concern (Fig 3a-3c, S9 Table). These fruits are both accessible and abundant during the fruit maturation period which often overlaps with the wet season in Trinidad from June to December. Field trials would help to better assess the competitiveness of the yeast in backyard environments in the presence of ripe fruits, including those assessed here and potentially other species. Many variables would have to be considered, for instance positioning of the bait around dwellings may still be impactful, particularly if the number of feeders is optimized. Another consideration is how effective application of these yeast baits may be during the bearing seasons during which these and other fruits could be a more important sugar source for mosquitoes. The significant differences observed for both *C. papaya* and *M. indica* also suggest that extracts from these fruits can be explored as an additive to further increase attraction to Sh.463-Soda bait stations.

The residual insecticidal activity of the Sh.463-Soda bait was particularly striking. When stored under simulated field conditions in the insectary and tested monthly over a 14-month period, the Sh.463-Soda bait continually induced high lethality in the four mosquito species tested (Fig 5). *C. quinquefasciatus* exhibited consistently high mortality throughout the study period, with 100% mortality recorded in 11 of 12 trials and 98.5% in one month (S11 Table). Other species, namely *A. aegypti*, *A. gambiae*, and *A. stephensi* also responded strongly (monthly mortality rates: 93–100%) at all timepoints tested, including at month 14 (S11 Table). This is particularly notable given that control of arboviral vectors like *A. aegypti* and *C. quinquefasciatus* has historically relied on repeated insecticide applications and is often hindered by resistance [51,52]. The sustained RNAi-induced lethality observed here suggests a viable, long-lasting alternative to conventional adulticidal strategies for these species. Similarly, the residual effect against the malaria vectors, *A. gambiae* and *A. stephensi*, is important, and confirms the potential of RNAi yeast-soda ATSBs as a potential complementary tool for malaria vector control. This is especially relevant in the context of residual malaria transmission insecticide resistance and the recent spread of the invasive vector, *A. stephensi*, in urban areas of Africa [3,53]. Overall, these results demonstrate that inactivated RNAi yeast remains stable and bioactive over extended periods, even under simulated field conditions, a critical property for products intended for deployment in tropical environments. Importantly, our findings support the potential feasibility of soda-baited RNAi treatments as effective, long-lasting and universally attractive mosquito control tools.

Finally, it should be noted that although the soda bottle feeders performed well (Fig 4), they were difficult to construct, and leaking could sometimes be observed. This may be due to the lower surface tension of soda (approximately 42.2 mN/m) compared to sugar water (73.5 mN/m) [54]. The design worked well enough for a prototype, but simplifying construction and correcting the leakage would be a helpful and seemingly straightforward next step, as an easy to construct low-maintenance feeder would be ideal.

## Conclusions

This study has demonstrated the potential for using soda-baited RNAi yeast as a potent and scalable platform for deploying ATSB-based mosquito control. The ability of the Sh.463-Soda formulation to induce high mortality across multiple species, combined with long-term residual activity, makes it a promising candidate, following further field testing in diverse climates, for potential future field-based implementation, particularly in resource-limited settings and as part of integrated vector management programs. Additionally, yeast-based formulations offer a cost-effective, environmentally friendly, species-specific alternative to chemical insecticides. The semi-field trials in Trinidad demonstrated that the RNAi yeast-soda bait may be competitive against some natural sugar sources. Future research should focus on further evaluation of the bait’s operational performance under field conditions for multiple mosquito vector species and in diverse geographical settings.

## Supporting information

S1 Table Efficacy of Sh.463 yeast RNAi biopesticide across five mosquito species using soda, sugar water and WH delivery baits. Values represent estimated marginal mean percentage mortality and 95% CI calculated from a binomial GLMM. The number of female mosquitoes tested was N = 225. Abbreviations: ASB = Attractive Sugar Bait for each respective category; Control = Control yeast +ASB. N/A means that 95% CIs could not be calculated because variance is zero in respective experimental groups.
(PDF)

S2 Table Mortality of mosquito species exposed to ASB-Soda, ASB-Sugar, and ASB-WH. The negative ASB control data are shown for ASB-Soda, ASB-Sugar and ASB-WH trials conducted alongside the experiments in Table 1. Mortality across the five mosquito species is expressed as mean ± SEM (%). The number of female mosquitoes tested was N = 225.
(PDF)

S3 Table Adult mosquito mortality across five species in dual-choice bioassays with different Sh.463 yeast sugar carriers. Values represent back-transformed model-estimated mortality percentages and their corresponding 95% (CIs). Parentheses indicate the alternative bait option available to the mosquitoes within each choice-test setup. The number of female mosquitoes tested was N = 225.
(PDF)

S4 Table Mosquito mortality rates for ASB-Soda and ASB-Sugar baits. Results from negative control trials on the efficacy of Sh.463 yeast RNAi biopesticide. Observed mosquito mean mortality rates for control experiments which were conducted in independent cages alongside dual choice experiments described in S1 Table. Mortality across the five mosquito species (N = 150 females per group) is expressed as mean ± SEM (%).
(PDF)

S5 Table Dual-choice feeding evaluation of Sh.463 biopesticide carriers using fluorescent dye tracking in *C. pipiens* (Uranine-marked Control yeast-Soda vs. Rhodamine-marked Control yeast-WH). Dual-choice tracer feeding assays were assessed using fluorescent markers (0.5% uranine green-colored and 0.5% rhodamine B red-colored) to validate carrier ingestion in dye-swap configurations. Mosquito ingestion was categorized by abdominal fluorescence visualization following a 4-hour feeding period. The indicated dye configuration used in S5 Table was swapped in S6 Table. Pairwise preference differences were evaluated using Chi-square goodness-of-fit tests (*χ*^2^ = 170.37, *df* = 1, *p* < 0.001, N = 200 mosquitoes). WH: Westham. Mixed = Ingestion of both bait formulations; N = Number of mosquitoes tested; *df* = Degrees of freedom.
(PDF)

S6 Table Dual-choice feeding evaluation of Sh.463 biopesticide carriers using fluorescent dye tracking in *C. pipiens* (Rhodamine-marked Control yeast-Soda vs. Uranine-marked Control yeast-WH). Dual-choice tracer feeding assays were assessed using fluorescent markers (0.5% uranine green-colored and 0.5% rhodamine B red-colored) to validate carrier ingestion in dye-swap configurations. Mosquito ingestion was categorized by abdominal fluorescence visualization following a 4-hour feeding period. The indicated dye configuration used in S6 Table was swapped in S5 Table. Pairwise preference differences were evaluated using Chi-square goodness-of-fit tests *χ*^2^ = 34.59, *df* = 1, p < 0.001 (N = 200 mosquitoes). WH: Westham. Mixed = Ingestion of both bait formulations; N = Number of observed mosquitoes; *df* = Degrees of freedom.
(PDF)

S7 Table Mosquito mortality rates for ASB-Soda and ASB-WH baits. Results from negative control trials on the efficacy of Sh.463 yeast RNAi biopesticide. Observed mosquito mean mortality rates for control experiments which were conducted in independent cages alongside experiments described in Fig 2. Mortality across the five mosquito species (N = 75 females per group) is expressed as mean ± SEM (%).
(PDF)

S8 Table Semi-field trials conducted on natural sugar sources only for three mosquito species in Trinidad. Results from feeding assays conducted on male (n = 15) and female (n = 15) mosquitoes for three replicates (N = 45) of the indicated species that were presented in flowers or fruits only trial cages. Average percentage mortality for triplicate experiments is expressed as mean ± SEM (%).
(PDF)

S9 Table Mosquito mortality and post-hoc pairwise comparisons under non-competitive (Sh.463 yeast only) and competitive (Choice) feeding regimes. Choice = Floral-fruit background + Sh.463 yeast. Significance values (*p*-values) were calculated using post-hoc pairwise Wald χ2 contrasts and adjusted for multiple testing using the Bonferroni correction, comparing Sh.463 yeast only and competitive feeding regimes within each species-sex-treatment cohort. Values represent % mortality with 95% confidence intervals in brackets. *p*-values indicate pairwise comparisons between Sh.463 yeast only and choice treatments. Model-estimated values are derived from a binomial GLM. Feeding assays were conducted on male (n = 15) and female (n = 15) mosquitoes for three biological replicate trials (N = 45).
(PDF)

S10 Table Overall 14-month residual insecticidal efficacy (mean % mortality) of soda-formulated Sh.463 yeast biopesticide across four mosquito species. Estimated marginal mean percentage mortality and 95% CIs are back-transformed from a GLMM. Control-Soda = Control yeast +Soda; Sh.463-Soda = Sh.463 yeast biopesticide delivered in soda formulation.
(PDF)

S11 Table Residual activity of Sh.463 RNAi yeast in soda over 14 months. Observed residual activity of Sh.463 RNAi yeast in soda was evaluated in the laboratory over a 14-month period. Mortality rates (mean %) for N = 2–6 trials per month are presented for Sh.463-Soda and Control-Soda across the different mosquito species and experimental time points.
(PDF)

S12 Table GLMM fixed effects for 14-month soda ATSB residual efficacy assays. Fixed effects evaluation performed using a GLMM with a binomial distribution and logit link function. Abbreviations: *F* = F-statistic ratio; *df*1 = Numerator degrees of freedom; *df*2 = Denominator degrees of freedom.
(PDF)
