# Supplementary material for: Soda-baited RNAi yeast insecticides as effective Attractive Targeted Sugar Baits (ATSBs) for mosquito control
Source: PLoS Negl Trop Dis. 2026 Sep 21;20(9):e0014319. doi: 10.1371/journal.pntd.0014319 (PMC13623123; doi:10.1371/journal.pntd.0014319)
Supplement: Supplementary file 1 [file pntd.0014319.s001.pdf]

S1 Table: Efficacy of Sh.463 yeast RNAi biopesticide across five mosquito species using soda, sugar water and WH delivery baits.

Species	Treatment Group	Soda Mean [95% CI]	Sugar Mean [95% CI]	WH Mean [95% CI]
A. aegypti	ASB	4.9 [2.6, 8.9]	4.6 [2.3, 9.0]	6.1 [3.5, 10.5]
	Control	3.1 [1.4, 6.6]	4.0 [1.9, 8.3]	9.1 [5.8, 14.0]
	Sh.463 yeast	98.2 [95.2, 99.4]	87.5 [81.5, 91.7]	96.3 [92.6, 98.2]
A. albopictus	ASB	1.8 [0.6, 4.8]	4.4 [2.4, 8.1]	3.7 [1.9, 7.2]
	Control	0.4 [0.1, 3.2]	3.1 [1.5, 6.5]	4.3 [2.3, 8.0]
	Sh.463 yeast	97.8 [94.6, 99.1]	99.1 [96.5, 99.8]	94.8 [90.9, 97.0]
A. gambiae	ASB	5.3 [2.9, 9.4]	1.3 [0.4, 4.1]	4.8 [2.5, 9.3]
	Control	3.5 [1.7, 7.1]	3.6 [1.8, 7.0]	5.8 [3.1, 10.4]
	Sh.463 yeast	100 N/A	100 [0.0, 100.0]	97.6 [93.9, 99.1]
C. pipiens	ASB	0.4 [0.1, 3.2]	1.8 [0.7, 4.7]	5.0 [2.8, 8.8]
	Control	0.9 [0.2, 3.6]	0.0 [0.0, 100.0]	3.0 [1.4, 6.3]
	Sh.463 yeast	94.7 [90.6, 97.1]	92.9 [88.6, 95.6]	89.4 [84.6, 92.8]
C. quinquefasciatus	ASB	0.0 N/A	1.6 [0.4, 6.3]	1.9 [0.7, 4.9]
	Control	0.0 N/A	1.6 [0.4, 6.3]	2.8 [1.3, 6.0]
	Sh.463 yeast	98.7 [95.9, 99.6]	100 [0.0, 100.0]	98.6 [95.8, 99.5]

Values represent estimated marginal mean percentage mortality and 95% CI calculated from a binomial GLMM. The number of female mosquitoes tested was N=225. Abbreviations: ASB = Attractive Sugar Bait for each respective category; Control =Control yeast +ASB. N/A means that 95% CIs could not be calculated because variance is zero in respective experimental groups.
