# Supplementary material for: Soda-baited RNAi yeast insecticides as effective Attractive Targeted Sugar Baits (ATSBs) for mosquito control
Source: PLoS Negl Trop Dis. 2026 Sep 21;20(9):e0014319. doi: 10.1371/journal.pntd.0014319 (PMC13623123; doi:10.1371/journal.pntd.0014319)
Supplement: Supplementary file 2 [file pntd.0014319.s002.pdf]

S2 Table: Results from negative control trials.

S2a: Mortality of mosquito species exposed to ASB-Soda, ASB-Sugar, and ASB WH.

		Mean Mortality rate ± SEM (%)
**Bait**	**Mosquito Species**	**ASB (N=225)**
**Soda**	*A. aegypti*	5.0 ± 1.00
	A. albopictus	1.8 ± 0.70
	A. gambiae	5.4 ± 1.32
	C. pipiens	0.4 ± 0.44
	C. quinquefasciatus	0
**10% Sugar**	*A. aegypti*	4.6 ± 1.36
	A. albopictus	4.4 ± 1.41
	A. gambiae	0.9 ± 0.57
	C. pipiens	2.2 ± 1.18
	C. quinquefasciatus	1.6 ± 1.79
**Westham bait**	*A. aegypti*	6.0 ± 2.00
	A. albopictus	3.6 ± 1.41
	A. gambiae	2.2 ± 3.38
	C. pipiens	4.4 ± 2.62
	C. quinquefasciatus	1.8 ± 0.97

The negative ASB control data are shown for ASB-Soda, ASB-Sugar and ASB-WH trials conducted alongside the experiments in Table 1. Mortality across the five mosquito species is expressed as mean ± SEM (%). The number of female mosquitoes tested was N=225.

S2b: Mosquito mortality rates for ASB-Soda and ASB-Sugar baits

Species	Mortality rate ± SEM (%)<br>ASB-Soda (N=75)	ASB-Sugar (N=75)
A. aegypti	6.0 ± 0.71	3.7 ± 1.43
A. albopictus	0.7 ± 0.67	0
A. gambiae	7.0 ± 3.37	0.8 ± 0.73
C. pipiens	5.0 ± 3.09	6.0 ± 2.83
C. quinquefasciatus	4.7 ± 2.40	2.0 ± 0.89

Observed mosquito mean mortality rates for control experiments which were conducted in independent cages alongside dual choice experiments described in Fig 2. Mortality across the five mosquito species (N=150 females per group) is expressed as mean ± SEM (%).

S2c: Mosquito mortality rates for ASB-Soda and ASB-WH baits

Species	Mortality rate ± SEM (%)<br>ASB-Soda (N=75)	ASB-WH (N=75)
A. aegypti	2.4 ± 0.98	0.8 ± 0.73
A. albopictus	6.7 ± 3.21	2.0 ± 0.89
A. gambiae	11.2 ± 2.29	10.7 ± 1.56
C. pipiens	1.7 ± 0.80	2.3 ± 1.17
C. quinquefasciatus	2.0 ± 2.19	3.2 ± 1.11

Observed mosquito mean mortality rates for control experiments which were conducted in independent cages alongside experiments described in Fig 2. Mortality across the five mosquito species (N=75 females per group) is expressed as mean ± SEM (%).
