# Supplementary material for: Soda-baited RNAi yeast insecticides as effective Attractive Targeted Sugar Baits (ATSBs) for mosquito control
Source: PLoS Negl Trop Dis. 2026 Sep 21;20(9):e0014319. doi: 10.1371/journal.pntd.0014319 (PMC13623123; doi:10.1371/journal.pntd.0014319)
Supplement: Supplementary file 3 [file pntd.0014319.s003.pdf]

S3 Table: Adult mosquito mortality across five species in dual-choice bioassays with different Sh.463 yeast sugar carriers.

Species	Choice-Test Setup / Sh.463 Yeast Carrier	Mean	Std. Error	95% CI (Lower)	95% CI (Lower)
A. aegypti	Soda (vs. WH)	87.4	0.02	82.8	90.8
	WH (vs. Soda)	14.9	0.031	9.8	22.1
	Sugar (vs. Soda)	22.1	0.038	15.4	30.6
A. albopictus	Soda (vs. WH)	81.4	0.024	76.2	85.6
	WH (vs. Soda)	15.5	0.031	10.3	22.8
	Sugar (vs. Soda)	26.9	0.042	19.5	35.9
A. gambiae	Soda (vs. WH)	93	0.014	89.5	95.4
	WH (vs. Soda)	22.8	0.036	16.5	30.6
	Sugar (vs. Soda)	54	0.047	44.7	63.1
C. pipiens	Soda (vs. WH)	95.3	0.013	91.9	97.3
	WH (vs. Soda)	6.2	0.02	3.2	11.4
	Sugar (vs. Soda)	9.3	0.026	5.3	15.9
C. quinquefasciatus	Soda (vs. WH)	86.4	0.021	81.7	90
	WH (vs. Soda)	1.2	0.009	0.3	4.9
	Sugar (vs. Soda)	15	0.032	9.6	22.7

Values represent back-transformed model-estimated mortality percentages and their corresponding 95% (CIs). Parentheses indicate the alternative bait option available to the mosquitoes within each choice-test setup. The number of female mosquitoes tested was N=225.
