# Supplementary material for: Soda-baited RNAi yeast insecticides as effective Attractive Targeted Sugar Baits (ATSBs) for mosquito control
Source: PLoS Negl Trop Dis. 2026 Sep 21;20(9):e0014319. doi: 10.1371/journal.pntd.0014319 (PMC13623123; doi:10.1371/journal.pntd.0014319)
Supplement: Supplementary file 5 [file pntd.0014319.s005.pdf]

S5 Table: Dual-choice feeding evaluation of Sh.463 biopesticide carriers using fluorescent dye tracking in *C. pipiens* (Uranine-marked Control yeast-Soda vs. Rhodamine-marked Control yeast-WH).

Feeding category	Abdomen Visual	Number Observed (N)	Proportion of Fed Population (%)
Control yeast-Soda only	Green	181	92.35
Control yeast-WH only	Red	3	1.53
Mixed	Red-Green	12	6.12
Total Fed	-	196	100

Dual-choice tracer feeding assays were assessed using fluorescent markers (0.5% uranine green-colored and 0.5% rhodamine B red-colored) to validate carrier ingestion in dye-swap configurations. Mosquito ingestion was categorized by abdominal fluorescence visualization following a 4-hour feeding period. The indicated dye configuration used in S5 Table was swapped in S6 Table. Pairwise preference differences were evaluated using Chi-square goodness-of-fit tests $\chi^2 = 162.39$, $df = 1$, $p < 0.001$ (N=200 mosquitoes). WH: Westham. Mixed = Ingestion of both bait formulations; N = Number of observed mosquitoes; *df* = Degrees of freedom.
