# Supplementary material for: Soda-baited RNAi yeast insecticides as effective Attractive Targeted Sugar Baits (ATSBs) for mosquito control
Source: PLoS Negl Trop Dis. 2026 Sep 21;20(9):e0014319. doi: 10.1371/journal.pntd.0014319 (PMC13623123; doi:10.1371/journal.pntd.0014319)
Supplement: Supplementary file 7 [file pntd.0014319.s007.pdf]

S7 Table: Mosquito mortality rates for ASB-Soda and ASB-WH baits

Species	Mortality rate ± SEM (%)	
	**ASB-Soda (N=75)**	**ASB-WH (N=75)**
A. aegypti	2.4 ± 0.98	0.8 ± 0.73
A. albopictus	6.7 ± 3.21	2.0 ± 0.89
A. gambiae	11.2 ± 2.29	10.7 ± 1.56
C. pipiens	1.7 ± 0.80	2.3 ± 1.17
C. quinquefasciatus	2.0 ± 2.19	3.2 ± 1.11

Results from negative control trials on the efficacy of Sh.463 yeast RNAi biopesticide. Observed mosquito mean mortality rates for control experiments which were conducted in independent cages alongside experiments described in Fig 2. Mortality across the five mosquito species (N=75 females per group) is expressed as mean ± SEM (%).
