# Supplementary material for: Soda-baited RNAi yeast insecticides as effective Attractive Targeted Sugar Baits (ATSBs) for mosquito control
Source: PLoS Negl Trop Dis. 2026 Sep 21;20(9):e0014319. doi: 10.1371/journal.pntd.0014319 (PMC13623123; doi:10.1371/journal.pntd.0014319)
Supplement: Supplementary file 8 [file pntd.0014319.s008.pdf]

S8 Table: Semi-field trials conducted on natural sugar sources only for three mosquito species in Trinidad.

	Average Mortality (%) ± SEM (%)		
**Species**	**Gender**	***A. cathartica***	**N = 45**
A. aegypti	Male	48.7 ± 8.09	n=3
A. albopictus	Male	73.3 ± 3.76	n=3
C. quinquefasciatus	Male	22.3 ± 9.60	n=3
A. aegypti	Female	51.1 ± 2.00	n=3
A. albopictus	Female	18.0 ± 5.86	n=3
C. quinquefasciatus	Female	15.3 ± 2.33	n=3
		C. roseus	
A. aegypti	Male	6.7 ± 3.76	n=3
A. albopictus	Male	48.7 ± 4.33	n=3
C. quinquefasciatus	Male	17.7 ± 7.86	n=3
A. aegypti	Female	2.3 ± 2.33	n=3
A. albopictus	Female	22.0 ± 5.86	n=3
C. quinquefasciatus	Female	4.7 ± 2.33	n=3
		H. rosa-sinensis	
A. aegypti	Male	49.0 ± 5.86	n=3
A. albopictus	Male	51.3 ± 15.45	n=3
C. quinquefasciatus	Male	42.3 ± 13.62	n=3
A. aegypti	Female	40.0 ± 0.0	n=3
A. albopictus	Female	9.0 ± 5.86	n=3
C. quinquefasciatus	Female	33.3 ± 17.35	n=3
		I. coccinea	
A. aegypti	Male	29.0 ± 14.64	n=3
A. albopictus	Male	26.7 ± 10.37	n=3
C. quinquefasciatus	Male	31.0 ± 17.35	n=3
A. aegypti	Female	40.0 ± 14.01	n=3
A. albopictus	Female	2.3 ± 2.33	n=3
C. quinquefasciatus	Female	29.0 ± 25.58	n=3
		L. camara	
A. aegypti	Male	2.3 ± 2.33	n=3
A. albopictus	Male	11.3 ± 8.09	n=3
C. quinquefasciatus	Male	11.0 ± 2.00	n=3

A. aegypti	Female	6.7 ± 6.67	n=3
A. albopictus	Female	0.0 ± 0.0	n=3
C. quinquefasciatus	Female	8.7 ± 4.33	n=3
		P. carica	
A. aegypti	Male	31.1 ± 21.78	n=3
A. albopictus	Male	44.3 ± 19.46	n=3
C. quinquefasciatus	Male	15.7 ± 12.33	n=3
A. aegypti	Female	2.3 ± 2.33	n=3
A. albopictus	Female	4.7 ± 2.33	n=3
C. quinquefasciatus	Female	20.3 ± 13.33	n=3
		M. indica	
A. aegypti	Male	0.0 ± 0.0	n=3
A. albopictus	Male	44.7 ± 9.60	n=3
C. quinquefasciatus	Male	11.0 ± 5.86	n=3
A. aegypti	Female	2.3 ± 2.33	n=3
A. albopictus	Female	26.7 ± 3.76	n=3
C. quinquefasciatus	Female	13.0 ± 0.0	n=3

Results from feeding assays conducted on male (n=15) and female (n=15) mosquitoes for three biological replicate trials (N=45) of the indicated species that were presented in flowers or fruits only trial cages. Average percentage mortality for triplicate experiments is expressed as mean ± SEM (%).
