# Supplementary material for: Soda-baited RNAi yeast insecticides as effective Attractive Targeted Sugar Baits (ATSBs) for mosquito control
Source: PLoS Negl Trop Dis. 2026 Sep 21;20(9):e0014319. doi: 10.1371/journal.pntd.0014319 (PMC13623123; doi:10.1371/journal.pntd.0014319)
Supplement: Supplementary file 9 [file pntd.0014319.s009.pdf]

S9 Table: Mosquito mortality and post-hoc pairwise comparisons under non-competitive (Sh.463 yeast only) and competitive (Choice) feeding regimes.

Species / Floral-fruit Background	Female Mortality (%)		Male Mortality (%)		Choice *p*-value	Sh.463 yeast Only *p*-value
	Choice	Sh.463 yeast Only	Choice	Sh.463 yeast Only		
A. aegypti						
A. cathartica	79	100	95	100	1	N/A
C. roseus	88	93	78	92	0.031	0.036
H. rosa-sinensis	70	100	84	100	0.021	N/A
I. coccinea	68	97	62	97	0.663	N/A
L. camara	59	95	50	96	0.031	0.312
C. papaya	18	92	28	97	< 0.001	0.501
M. indica	40	94	21	91	0.505	0.501
A. albopictus						
A. cathartica	48	85	81	98	< 0.001	0.002
C. roseus	96	86	92	81	0.163	0.777
H. rosa-sinensis	93	100	96	100	0.645	N/A
I. coccinea	76	84	70	86	0.633	0.56
L. camara	86	91	80	91	0.788	0.396
C. papaya	77	96	85	98	0.267	0.303
M. indica	66	85	41	77	< 0.001	< 0.001
C. quinquefasciatus						
A. cathartica	100	100	100	100	N/A	N/A
C. roseus	88	73	71	60	< 0.001	0.271
H. rosa-sinensis	100	100	100	100	N/A	N/A
I. coccinea	98	99	97	99	N/A	0.073
L. camara	72	88	55	85	0.167	0.174
C. papaya	47	91	52	95	0.391	N/A
M. indica	58	88	27	76	0.047	0.366

Choice = Floral-fruit background + Sh.463 yeast. Significance values (*p*-values) were calculated using post-hoc pairwise Wald $\chi 2$ contrasts and adjusted for multiple testing using the Bonferroni correction, comparing Sh.463 yeast Only and competitive feeding regimes within each species-sex-treatment cohort. Values represent % mortality with 95% confidence intervals in brackets. *p*-values indicate pairwise comparisons between Shaker Only and Choice treatments. Model-estimated values are derived from a binomial GLM. Feeding assays were conducted on male (n=15) and female (n=15) mosquitoes for three biological replicate trials (N=45).
