# Supplementary material for: Soda-baited RNAi yeast insecticides as effective Attractive Targeted Sugar Baits (ATSBs) for mosquito control
Source: PLoS Negl Trop Dis. 2026 Sep 21;20(9):e0014319. doi: 10.1371/journal.pntd.0014319 (PMC13623123; doi:10.1371/journal.pntd.0014319)
Supplement: Supplementary file 10 [file pntd.0014319.s010.pdf]

S10 Table: Overall 14-month residual insecticidal efficacy (mean % mortality) of soda-formulated Sh.463 yeast biopesticide across four mosquito species.

Mosquito Species	Treatment	Mean (%)	95% CI Lower (%)	95% CI Upper (%)
C. quinquefasciatus	Control-Soda	0.40	0.10	1.70
	Sh.463-Soda	99.90	99.20	100.00
A. stephensi	Control-Soda	4.20	1.30	13.10
	Sh.463-Soda	100.00	0.00	100.00
A. aegypti	Control-Soda	8.10	1.90	29.20
	Sh.463-Soda	98.80	93.40	99.80
A. gambiae	Control-Soda	2.20	0.30	14.20
	Sh.463 -Soda	100.00	0.00	100.00

Estimated marginal mean percentage mortality and 95% Cis are back-transformed from a GLMM. Control-Soda = Control yeast +Soda; Sh.463-Soda = Sh.463 yeast biopesticide delivered in soda formulation.
