# Supplementary material for: Soda-baited RNAi yeast insecticides as effective Attractive Targeted Sugar Baits (ATSBs) for mosquito control
Source: PLoS Negl Trop Dis. 2026 Sep 21;20(9):e0014319. doi: 10.1371/journal.pntd.0014319 (PMC13623123; doi:10.1371/journal.pntd.0014319)
Supplement: Supplementary file 11 [file pntd.0014319.s011.pdf]

S11 Table: Residual activity of Sh.463 RNAi yeast in soda over 14 months

		Mortality rate ± SEM (%)	
Month	Species	Control-Soda	Sh.463-Soda
1	*A. stephensi*	3.0 ± 0.0	99.3 ± 1.50
	C. quinquefasciatus	0	98.5 ± 1.06
2	*C. quinquefasciatus*	0	100
	A. stephensi	0.0 ± 0.0	100
3	*C. quinquefasciatus*	0	100
4	*A. aegypti*	8.34 ± 1.67	100
	C. quinquefasciatus	0	100
5	*C. quinquefasciatus*	0	100
6	*C. quinquefasciatus*	0	100
7	*C. quinquefasciatus*	0	100
8	*C. quinquefasciatus*	0	100
10	*A. aegypti*	1.0 ± 1.00	93.0 ± 4.12
	A. gambiae	0.7 ± 0.67	100
	A. stephensi	2.3 ± 0.75	100
	C. quinquefasciatus	14.0 ± 2.00	100
11	*C. quinquefasciatus*	0	100
12	*C. quinquefasciatus*	1.9 ± 1.90	100
13	*A. aegypti*	10.0 ± 2.00	100
	A. stephensi	6.0 ± 2.00	100
	C. quinquefasciatus	0	100
14	*A. gambiae*	4.5 ± 1.50	100
	A. stephensi	6.0 ± 2.00	100

C. quinquefasciatus	0	100

Note: Observed residual activity of Sh.463 RNAi yeast in soda was evaluated in the laboratory over a 14-month period. Mortality rates (mean %) for n=2–6 trials per month are presented for Sh.463-Soda and Control-Soda across the different mosquito species and experimental time points.
