# Supplementary material for: Soda-baited RNAi yeast insecticides as effective Attractive Targeted Sugar Baits (ATSBs) for mosquito control
Source: PLoS Negl Trop Dis. 2026 Sep 21;20(9):e0014319. doi: 10.1371/journal.pntd.0014319 (PMC13623123; doi:10.1371/journal.pntd.0014319)
Supplement: Supplementary file 12 [file pntd.0014319.s012.pdf]

S12 Table: GLMM fixed effects for 14-month soda ATSB residual efficacy assays

Source	*F*	*df*1	*df*2	p-value
Mosquito Species	0.020	3	103	0.996
Sh.463 yeast	0.063	1	103	0.802
Mosquito Species * Sh.463 yeast	6.213	3	103	0.001
Calendar Month	0.028	1	103	0.868

Fixed effects evaluation performed using a GLMM with a binomial distribution and logit link function. Abbreviations: *F* = F-statistic ratio; *df*1 = Numerator degrees of freedom; *df*2 = Denominator degrees of freedom.
